# Pathogenesis of allergic diseases and implications for therapeutic interventions

**DOI:** 10.1038/s41392-023-01344-4

**Published:** 2023-03-24

**Authors:** Ji Wang, Yumei Zhou, Honglei Zhang, Linhan Hu, Juntong Liu, Lei Wang, Tianyi Wang, Haiyun Zhang, Linpeng Cong, Qi Wang

**Affiliations:** 1 National Institute of TCM constitution and Preventive Medicine, School of Chinese Medicine, Beijing University of Chinese Medicine, Beijing, 100029 P.R. China; 2 National Cancer Center/National Clinical Research Center for Cancer/Cancer Hospital, Chinese Academy of Medical Sciences and Peking Union Medical College, Beijing, 1000210 China

**Keywords:** Immunological disorders, Immunological disorders

## Abstract

Allergic diseases such as allergic rhinitis (AR), allergic asthma (AAS), atopic dermatitis (AD), food allergy (FA), and eczema are systemic diseases caused by an impaired immune system. Accompanied by high recurrence rates, the steadily rising incidence rates of these diseases are attracting increasing attention. The pathogenesis of allergic diseases is complex and involves many factors, including maternal-fetal environment, living environment, genetics, epigenetics, and the body’s immune status. The pathogenesis of allergic diseases exhibits a marked heterogeneity, with phenotype and endotype defining visible features and associated molecular mechanisms, respectively. With the rapid development of immunology, molecular biology, and biotechnology, many new biological drugs have been designed for the treatment of allergic diseases, including anti-immunoglobulin E (IgE), anti-interleukin (IL)-5, and anti-thymic stromal lymphopoietin (TSLP)/IL-4, to control symptoms. For doctors and scientists, it is becoming more and more important to understand the influencing factors, pathogenesis, and treatment progress of allergic diseases. This review aimed to assess the epidemiology, pathogenesis, and therapeutic interventions of allergic diseases, including AR, AAS, AD, and FA. We hope to help doctors and scientists understand allergic diseases systematically.

## Introduction

Allergic diseases are systemic disorders caused by an impaired immune system. Different allergic diseases, including AR, AAS, AD, FA and eczema, are caused by complex interactions between genetic and environmental factors. Allergic diseases are listed by the World Health Organization (WHO) as one of the top three disorders to be prevented and controlled in the 21st century. An allergic disease, whilst a systemic disease, can also manifest as different local maladies, all of which may lead to anaphylactic shock in severe cases. The incidence of allergic diseases is high, bringing much suffering to patients. It is estimated that nearly 500 million and 300 million individuals worldwide have AR and AAS, respectively,^1^ with an increasing number of cases. For AAS, mortality rates in women and men are 90 and 170 per million individuals, respectively. About 96% of asthma deaths occur in low-and-middle-income countries.^2^ It is currently estimated that FA affects 1–10% of the total population.^3^ The global prevalence rate of AD is 8%,^4,5^ with a lifetime prevalence reaching 20%.^6^ In 2019, there were 171.17 million patients worldwide with AD.^7^

Due to the different sites of allergic diseases, the clinical and pathological manifestations also differ. In AR, after stimulation by allergens, including airborne dust mites associated with fecal particles, cockroach remains, pet dander, molds and pollens, inflammatory cells such as mast cells, CD4^+^ T cells, B cells, macrophages and eosinophils infiltrate the lining of the nasal cavity, with infiltration into the nasal mucosa. T helper 2 (Th2) cells promote the release of immunoglobulin and cytokines, including interleukin (IL)-3, IL-4, IL-5, and IL-13; meanwhile, IgE is also produced by plasma cells. There is, however, still some uncertainty around the source of IgE production. Follicular helper T (Tfh) cells are a subpopulation of CD4^+^ T-effector cells, and in recent years it has been discovered that the key cells regulating IgE production are not Th2 cells, but Tfh cells. Allergens cross-link IgE that interact with mast cells, which further induces the release of multiple mediators (including histamine and leukotrienes), promotes arteriole dilation and vascular permeability, and causes pruritus, runny nose, mucus secretion, and pulmonary smooth muscle contraction.^8^ Over the next 4–8 hours, the released mediators and cytokines induce subsequent cellular inflammatory reactions (late inflammatory response), leading to the recurrence of symptoms, often nasal congestion, which generally persist.^8,9^ The immunopathological profiles of AR and AAS are very similar in terms of eosinophil, mast cell and Th2 cell infiltration. Although structural changes in airway remodeling are well characterized in AAS, they may also occur in AR. There are also pathophysiological differences between AR and AAS. In the AAS disease, mucosal pathological alterations comprise epithelial hyperplasia, goblet cell metaplasia and increased mucus generation. In the submucosal layer, smooth muscle hypertrophy, collagen accumulation and large mucus glands prevail, leading to airway narrowing and enhanced mucus generation during an asthma attack,^10^ with symptoms such as difficulty breathing, wheezing, chest pain, and coughing.^11^ The pathogenesis of AD is mainly reflected by a complex interplay between epidermal barrier dysfunction, abnormal skin microbiota and dysregulated type 2 T cell immunity.^12,13^ The above pathogenesis induces a series of pathological manifestations such as filamentous aggregation; weak skin barrier due to protein shortage promoting inflammatory reactions and T cell infiltration; *S. aureus* colonization or infection disrupting the skin barrier and inducing an inflammatory response as well as the development of epidermal edema (“spongy sclerosis”); local Th2 immune reactions further reducing the barrier function, promoting dysregulation that favors *Staphylococcus* species, especially *S. aureus*, which triggers pruritus. FA is an IgE-dependent type I hypersensitivity to a specific food allergen. Its pathological process is divided into two stages: in the allergic sensitization stage, the initial exposure to the allergen results in tolerance breakdown, with subsequent generation of specific IgE, vasoactive substances and allergic response mediators such as histamine and platelet activating factor.^14^ During the provocation phase, degranulation of effector cells, such as mast cells, induces allergic inflammation, and serotonin or 5-hydroxytryptamine is released in large amounts, resulting in acute gastrointestinal symptoms, including diarrhea. Besides mast cells, an allergen also reacts with sensitized basophils in the circulation, triggering a life-threatening systemic reactions featuring multiple-organ and system involvements, hypotension and shock.^15^ After repeated exposure to food allergens, persistent allergic inflammatory reactions occur and tissue mast cells increase, resulting in persistent gastrointestinal reactions.^16^

The pathogenesis of allergic diseases is complex, involving many factors such as genetics, epigenetics, environmental factors, microecology and the body’s immune function. Their recurrence rate is high, which brings great pain to and imposes a severe financial burden on patients. Therefore, this manuscript comprehensively analyzes allergic diseases, from a brief introduction of their history to their mechanism and treatment, hoping to provide not only a systematic understanding of such diseases, but also a reference for clinical doctors and scientists.

## A brief history of allergic diseases

Since ancient times, people have always been drawn to allergic diseases, studying their pathogenesis and developing related treatments. The earliest recorded allergic reaction in human history was the death of the Egyptian pharaoh Menes after being bitten by a bumblebee around 2641 BCE.^17^ Theories on the actual causes and diagnosis of allergic diseases were further developed in the 19^th^ century, precisely in 1819, when the British physician John Bostock, at the Royal Society of Reported Medicine, attributed summer eye and nose discomfort to hay, naming the condition “hay fever”.^18^ Later in 1868, Eosinophilia was first observed by Henry Hyde Salter, in the sputum of a patient with an allergic disease.^19^

**Fig. 1:**
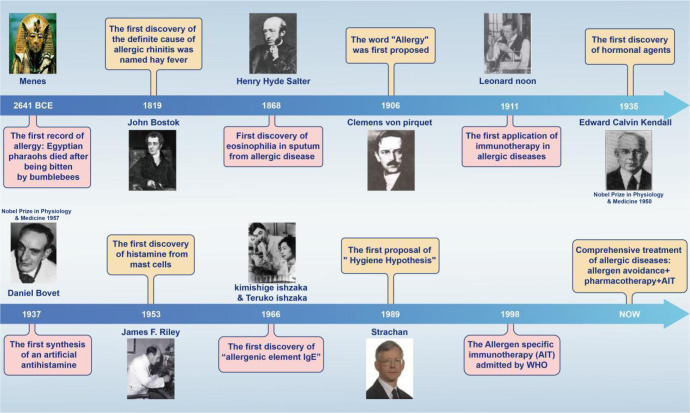
Timeline of major findings related to allergic diseases. Allergic reactions in Western countries were first recorded in 2641 BCE, when the Egyptian Pharaoh Menes died after being bitten by a bumblebee. In 1911, Leonard Noon published an article in *The Lancet*, reporting a clinical paper treating pollinosis by subcutaneous injection of grass pollen extract, which marked the beginning of modern immunotherapy. In 1966, the Japanese scientist Ishizaka and his wife discovered IgE, which led to a leap in the understanding of immediate allergy. Following their discovery, IgE became a new indicator for the diagnosis of allergy

The pathogenesis and treatment of allergic diseases have made rapid progress in the 20^th^ century. The word “allergy” was coined by Clemens von Pirquet in 1906,^20,21^ which is considered to be the beginning of modern allergy science. In 1911, Leonard Noon was the first to be successful in the treatment of pollen-associated AR with low-dose flower infusion, setting a precedent for immunotherapy.^22^ Edward Calvin Kendall discovered the adrenocortical hormone and determined its structure and physiological effects in 1935, earning the Nobel Prize in Physiology or Medicine in 1950.^23^ Daniel Bovet synthesized antihistamines in 1937 and earned the Nobel Prize in Physiology or Medicine in 1957, which brought hope in the treatment of allergic diseases and has been in clinical use to this day.^24,25^ In 1953, James F. Riley was the first to discover that histamine in the human body mainly comes from mast cell granules.^26^ Up to this point in history, basic treatment methods for allergic diseases had been established, but no breakthrough had been made in mechanistic research. In terms of pathogenesis, the Ishzaka couple discovered in 1966 that the reactive hormone in the serum of patients with allergic diseases was IgE,^27^ providing a new experimental tool and concept for serological research. In 1989, the epidemiologist Strachan proposed the “hygiene hypothesis” on the basis that “compared with an only child, children in large families have a lower risk of developing pollen allergy and eczema”.^28^ The hygiene hypothesis has laid a solid foundation for studying the pathogenesis of allergic diseases from the perspectives of modern immunology, microecology, and antibiotic application. Current guidelines suggest a combined application of allergen avoidance, pharmacotherapy, and/or allergen-specific immunotherapy (AIT)^29,30^ (Fig. 1).

## Mechanism

### Genetics and epigenetics

#### Gene-environment interactions in allergy diseases

Allergic disease is a complex disorder, whose etiology and development may involve genetic and environmental factors. Although the innate and adaptive immune systems are critical in regulating the adaptation to the external microenvironment,^31^ allergic diseases are considered a major cause of immune dysfunction caused by the interactions of multiple genes and the external environment in cells.^32,33^

**Fig. 2:**
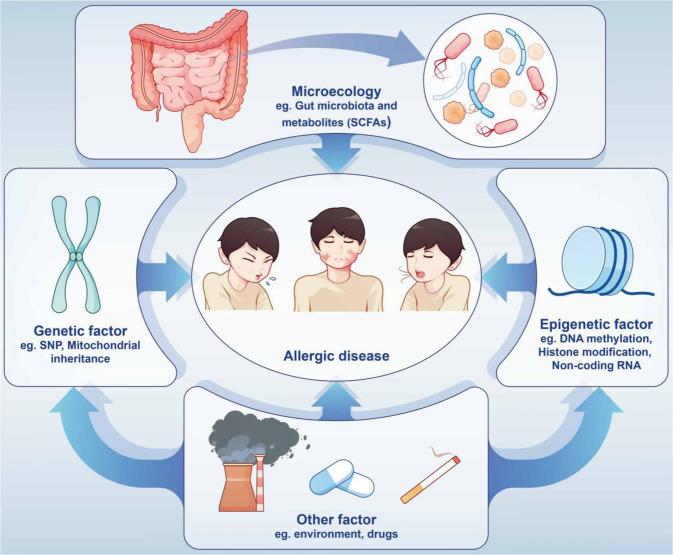
Allergic diseases are caused by a variety of factors. External factors include changes in gut microbiota and metabolites, drugs, and air pollution. Internal influencing factors include genetic and epigenetic changes

The research boom in genetics and epigenetics has substantially promoted research progress for allergic diseases. The application of genome-wide association studies (GWASs), single nucleotide polymorphism (SNP) analysis, and epigenome-wide association studies (EWASs) has laid a solid foundation for exploring the genetics of allergic diseases. Epigenetic studies mainly focus on DNA methylation, post-translational histone modifications, and non-coding RNAs. Epigenetics can explain the occurrence and development of allergic diseases in the external environment from various aspects, elucidate the mechanism of immune response plasticity in allergic disorders, and even provide diagnostic biomarkers and therapeutic targets for allergic disorders^34,35^ (Fig. 2).

#### SNPs and related GWASs in allergic diseases

When the Human Genome Project was completed in 2001,^36^ scientists were surprised to find that most genome sequence variations involve SNPs. SNP diversity can be found throughout different regions of the genome,^37^ including introns, exons, promoters, enhancers and intergenic regions, with SNPs considered as the basis of DNA sequence variation.^38^

##### Hereditary studies

Both genetics and the environment are critical to the etiology and development of allergic diseases, and it is difficult to distinguish their independent roles in allergic diseases when they are combined. Twins’ studies can be used to separate genetics from environmental factors, providing clues to the genetic component of allergic diseases. In fact, research into the genetics of allergic disease also began in twins. Twin studies revealed that FA had high heritability, and GWAS and candidate gene studies indicated marked associations of the human leukocyte antigen(HLA)-DR and HLA-DQ region with genetic variants in multiple genes, including Filaggrin (FLG), the HLA locus, and Forkhead Box Protein P3 (FOXP3).^39^ Peanut allergy in 82% of identical twins far exceeds the 20% concordance rate observed in dizygotic twins,^40,41^ further supported by the fact that children whose parents or siblings have peanut allergy are 7 times more likely to develop the disease compared with children without family risk factors. In general, heritability estimates for FA are as high as 81%,^42^ while the heritability of AR is estimated at approximately 91%.^42^ Twin studies reveal that about 25% of phenotypic variation in asthma severity can be explained by genetic factors; for example, RAD50- IL13 on chromosome 5q and the ORMDL sphingolipid biosynthesis regulator 3 (ORMDL3)- gasdermin B (GSDMB) locus on chromosome 17q21 were found to be associated with asthma severity.^43^ Besides, the concordance rate for AD in identical twins is about 80%, which is remarkably elevated compared with the 20% found in fraternal twins.^44^

##### SNP and GWAS analyses in allergic diseases

To date, SNPs in no less than 34 loci and 46 genes are considered to have AD risk in different populations around the world.^45^ Loss-of-function mutations in FLG, which encodes filaggrin (a skin barrier protein), are considered the most important genetic risk factor for AD, although variants affecting skin and systemic immune reactions also play critical roles.^46^ In an AAS disease study, after analysis by GWAS, Sarnowski et al. recently detected five genetic variants related to age at onset in 5,462 asthma cases, at or around recombinant Cylindromatosis (CYLD) on 16q12 (rs1861760), IL1RL1 on 2q12 (rs10208293), HLA-DQA1 on 6p21 (rs9272346), IL33 on 9p24 (rs928413) and GSDMA on 17q12 (rs9901146), with the last four also showing associations with susceptibility to allergic diseases. Recombinant Mucin 5 Subtype AC (MUC5AC) is considered an essential factor in the natural barrier function of the airway and has a potential association with moderate to severe asthma.^47^ Studies have shown that SNP changes of ORMDL3 and the TSLP promoter gene are involved in AAS;^48,49^ polymorphisms in the alpha chain coding region of IL-4 receptor are also associated with AAS.^50^ Susceptibility-related GWAS data showed that asthma-related IL-33 genes are all associated with asthma and AR.^51,52^ Changes in IL-4 gene single nucleotide polymorphisms can increase the risk of AR;^52^ individuals with the Vitamin D (1,25- dihydroxyvitamin D3) receptor (VDR) rs2228570 CC and vitamin D-binding protein (VDBP) rs7041 GG genotypes have a high risk of asthma progression.^53^ In FA analysis, peanut allergy is clearly associated with specific locus changes in the HLA-DR and HLA-DQ genes.^54^ Molecular genetic analysis of the GG, GA, and AA genotypes of the IL-13 R130Q gene polymorphism revealed markedly elevated incidence rates of the GA and AA genotypes in comparison with healthy control individuals.^55^ Besides, the serpin B serpin (SERPINB) and cytokine gene clusters increase the risk of any FA, as well as the C11orf30/LRRC32 locus.^56^

In the study of AR, the largest GWAS revealed 20 novel loci associated with AR risk,^57^ including IL7R at 5p13.2 and SH2B adaptor protein 3 (SH2B3) on chromosome 12q24.12, which separately participate in V(D)J recombination of T cell and B cell receptors,^58^ blood eosinophil count^59^ and T cell activation pathways.^60^ Furthermore, we noted that AR risk loci have important effects on innate and adaptive immune responses. Loci near C-X-C chemokine receptor type 5 (CXCR5) on 11q23 and Fc Fragment of IgE Receptor Ig (FCER1G) on 1q23.3 separately encode chemokine receptor in B cells and follicular T cells^61^ and the γ chain of IgE receptor.^62^ Broad-complex,tramtrack and bric-a-brac and cap’n’collar homology 2 (BACH2) on 6q15 is critical to the induction immunomodulatory function of memory B and T cells,^63,64^ Leukocyte tyrosine kinase (LTK) and TYRO3 protein tyrosine kinase (TYRO3) modulate Th2-type immune responses; RAR-related orphan receptor A (RORA) regulates the development and inflammatory response of Th2 innate lymphocytes, and tumor necrosis factor (ligand) superfamily, member 11 (TNFSF11) is involved in dendritic cell activation of T cells. The Viral protein R binding protein (VPRBP) gene controls T cell proliferation and contributes to V(D)J recombination in B cells.^65–68^ Surprisingly, the frequencies of the tumor necrosis factor-α (TNF-α) and MRPL4 genes are starkly elevated in AR cases in Han individuals^69^ (Table 1).

**Table 1:** Summary of genomic loci of allergic disease

Chromosome	Gene	SNP (Variant)	Possible Allergic Mechanism	Related disease	References
6p21.32	HLA-DR, -DQ	rs7192, rs9275596	Antigen-specific immune response	FA	^529^
18q21.3	SERPINB	rs12123821	Immunological regulation or epithelial barrier function	FA	^56^
19p13	MRPL4	rs8111930	Involved in inflammatory adhesion process	AR	^530^
11q13	C11orf30/LRRC32	rs2155219	Epithelial barrier function, regulatory	AR	^531^
			T-cell function, and immune tolerance		
4p14	TLR6	rs3860069	Pattern recognition receptors in innate immunity	AR	^531^
5q31.1	IL4	rs2243250	Promotes isotype class switching from IgM to IgE, promote the differentiation of T cells to a Th2 cell phenotype	AR with Asthma	^532^
5q31.1	IL13	rs20541			
5q22.1	TSLP	rs1898671	Epithelial barrier function, regulatory	AR	^531^
			T-cell function, and immune tolerance		
4q27	IL2	rs17454584/a	Immune regulatory effects.	AR	^54^
3p21.2	VPRBP	rs62257549	Involved in T cell proliferation and V(D)J recombination in B cell development.	AR	^54^
11q12-13	FCER1B	rs569108, rs512555	IgE cross-links allergen-bound receptors	AR	^533^
13q14.11	TNFSF11	rs7328203	Involved in hematopoiesis and downstream of T cell receptor activation.	AR	^54^
12q24.12	SH2B3	rs35350651			
3p21.2	VPRBP	rs62257549	Involved in T cell proliferation and V(D)J recombination in B cell development	AR	^54^
15q22.2	RORA	rs1051906	Involved in development of natural helper cell.	AR	^54^
10q24.32	ACTR1A; TMEM180	rs35597970	Involved in regulating TLR-4 and cytokine signaling	AR	^54^
5p13.2	IL7R	rs7717955	V(D)J recombination of B and T cell receptors;T cell subtypes have different levels of IL-7R on the cell surface	AR	^54^
1p31.1	LRRIQ3; NEGR1	rs2815765	Cell adhesion	AR	^54^
17q21.2	GSDMB	rs7216389	Terminal differentiation of epithelial cells	AAS	^534^
5p13.3	CD14 (-159 C/T)	rs2569190	Monocyte activity	AAS	^535^
17q11.2	NOS2	rs10459953	Expressed on T cells, macrophages, epithelial cells, mast cells, eosinophils, and neutrophils in response to inflammatory stimuli	AAS	^536^
Xp11.23	FOXP3	rs3761548,rs3761549	Transcription factor of Treg cell	AAS	^537^
5q31	KIF3A	rs11740584, rs2299007	Increased transepidermal water loss (TEWL)	AD	^538^
21q22.23	UBASH3A	rs11203203	A phosphatase that regulates the T cell receptor (TCR) signaling pathway	AD	^539^
1q12	FLG	rs7927894	Epidermal barrier	AD	^540^
5q21.3	TSLP	rs11466749	Genes encoding alarmins produced by keratinocytes		
17q21.2	ORMDL3	rs7216389	Epidermal barrier		
6p21.3	GPSM3	rs176095		AD	^541^
11p15.4	NLRP10	rs878860	Encodes a protein that belongs to the NALP protein family but lacks the leucine-rich repeat region	AD	^541^
3p21.33	GLB1	rs6780220	Encodes β-galactosidase-1	AD	^541^
3q13.2	CCDC80	rs12634229	Encodes a protein involved in the induction of C/EBPα and peroxisome proliferator-activated receptor γ (PPARγ)	AD	^541^
7p22	CARD11	rs4722404	An protein for T-cell receptor (TCR) and B-cell receptor (BCR) signaling	AD	^541^
10q21.2	ZNF365	rs10995251	Encodes a T-cell anergy-associated transcription factor	AD	^541^
20q13	CYP24A1-PFDN4	rs16999165	Encodes a mitochondrial cytochrome P450 superfamily enzyme	AD	^541^

#### Mitochondrial inheritance in allergic disease

Maternal inheritance is considered the most critical player in allergic disease occurrence. Most offspring mitochondria are inherited from the mother, and mitochondrial inheritance is tightly associated with asthma occurrence.^70–73^ Mitochondrial DNA (mtDNA) variants are significantly associated with allergic disorders, including AD and asthma. A report demonstrated that in 69 mtDNA variants, the rs28357671 locus of the MT-ND6 gene was significantly associated with mitochondrial function genes in allergic diseases, including: NLR Family Member X1 (NLRX1), oculocutaneous albinism II (OCA2) and coiled-coil-helix-coiled-coil-helix domain containing 3 (CHCHD3).^74^ Interestingly, the genetic cause of asthma in females may be associated with a dysfunction of mitochondrial MT-ND2 and MT-RNR2 genes, while in males, mutations in the mitochondrial cytochrome-b (CYB) gene leads to changes in reactive oxygen species (ROS) and asthma development.^75^

Mutations affecting mitochondrial tRNA genome sequences have been observed in the placenta of asthmatic mothers and are associated with AAS;^76^ for example, a rare mutation in the A3243G-tRNA Leu (UUR) MELAS gene, which is thought to be associated with asthma, was found in asthmatic patients. Maternally inherited mitochondrial diseases have been reported.^77^ Fukuda’s team found that 9 of the 13 differentially expressed genes in allergic patients were mitochondria-related genes, including those producing cytochrome oxidases II and III, and NADH dehydrogenase.^76,78,79^ In addition, polymorphisms in the ADAM metallopeptidase domain 33 (ADAM33) and cytochrome b genes located on chromosome 20 have been associated with asthma susceptibility, both of which are closely associated with mitochondrial oxidative function.^80^ Mitochondrial haploidy and elevated serum IgE amounts are associated in Europeans,^73^ which may involve diverse mutations in genes that encode mitochondrial tRNAs.^76^ The ATP synthase mitochondrial F1 complex assembly factor 1 gene was implicated in asthma in Caucasian children.^81^ Some AAS is closely related to mtDNA deficiency, and alterations in more than 25 genes (ORMDL3, 2PBP2, GSDMB, PDE4D, VEGF, Wnt, MMP-12, PRKCA, JAG1, ANKRD5, TGF-β1, IL-12β, IL-10, IL-13, IL-17, IL-25, and β2-adrenergic receptors) are associated with abnormal changes in the immune system in AAS.^80,82–84^

#### Epigenetics and Epigenome-wide Association Study (EWAS)-related analysis of allergic diseases

Epigenetics are heritable features that affect gene expression without altering the DNA sequence.^85^ DNA methylation is an effective factor to distinguish allergic patients from healthy people.^44^ DNA methylation is reflected by a methyl group added to cytosine at position 5 by DNA methyltransferases to form 5-methylcytosine,^86^ where a cytosine nucleotide is called a CpG, followed by a guanine nucleotide.^87^ CpG islands typically contain more than 200 bases, of which more than 60%-80% are guanines and cytosines (G + C).^88^ Methylation of CpG islands at transcription start sites (TSSs) of genes leads to gene activation or repression, and is generally thought to repress gene transcription.^89^

A cross-sectional study found that DNA methylation of allergy-related genes in the whole blood of allergic children may be a common parameter affecting asthma, rhinitis, and eczema; a total of 21 differential CpG loci were screened, 10 of which were in the pulmonary epithelium. The sites of replication, related to acyl-CoA thioesterase 7 (ACOT7), Lectin, Mannose Binding 2 (LMAN2) and Claudin 23 (CLDN23) genes, were all derived from eosinophils.^90^ Thus, changes in eosinophil levels are reflected by changes in methylation, unveiling a possible mechanism for phenotypic alterations in immune response-associated features.

In addition, methylation plays an important role in AD pathogenesis. The TSLP gene promoter is hypomethylated in the damaged skin of AD patients.^91^ Methylation is not specific to DNA, but is closely related to disease. Demethylation of histone H3 residues in the FOXP3 gene promoter region and hypermethylation of histone H3 residues in the RORC gene promote the differentiation of Th0 cells towards a regulatory T (Treg) phenotype. Conversely, these events cause Treg deficiency, one of the hallmarks of AD pathogenesis.^34,92–95^ Furthermore, CpG hypermethylation in IL-4 is negatively correlated with serum total IgE levels, explaining the role of Th2 immunity in AD.^96^ Enhanced hypermethylation of S100 calcium binding protein A5 (S100A5) was found in the epidermal part of lesions in AD cases in comparison with healthy individuals.^97^ Hypomethylation was observed in Recombinant Keratin 6 A (KRT6A) in keratinocytes, and methylation of cg07548383 in FLG also elevates AD risk.^98^

DNA methylation is also critical for AAS pathogenesis, occurrence, and development. An EWAS detected a total of 40,892 CpG sites methylated in important genes C-C motif chemokine ligand 26 (CCL26, a chemokine) and mucin 5 AC (MUC5AC, a mucin with airway defense function) among AAS patients compared with control cases.^99^ Chromosome 17q12-q21 hypermethylation contributes to asthma pathogenesis, with regulatory effects on all five protein-coding genes of this region, including IKAROS family zinc finger 3 (Aiolos) (IKZF3), zona pellucida-binding protein 2 (ZPBP2), ORMDL3, gasdermin A (GSDMA) and GSDMB.^100^ The STAT5A gene is hypermethylated in the 17q21.2 region and has been linked to increased Th1 responses and reduced infiltration of eosinophils in the airway epithelium.^101^ In childhood asthma, cg23602092 gene methylation status was linked to asthma symptoms,^102^ and hypomethylation of arachidonate 15-lipoxygenase (ALOX15) gene 17p13.2 and periostin, osteoblast specific factor (POSTN) gene 13q13.3 in nasal epithelial cells is associated with increased Th2 function.^103^ Methylation sites in multiple white blood cell (WBC) genes show significant associations with total IgE amounts, with the two most significant genes (ACOT7 and ZFPM1) associated with asthma.^104^ In adults, WNT2 gene hypermethylation in the 7q31.2 region in blood specimens is involved in neutrophilic asthma,^105^ and ORMDL3 hypermethylation in endobronchial airway epithelial cells contributes to asthma.^106^ Following hypermethylation at CpG sites, FOXP3 (12q15) and interferon-γ (IFN-γ) (Xp11.23) lead to altered T cell function and repressed Treg and T effector cell-related genes in blood.^107^ In adolescents, interleukin-5 receptor alpha (IL-5RA) (3p26.2) hypomethylation in blood was linked to asthma.^108^

In the AR disease, DNA methylation levels are tightly associated with CD4^+^ T cell amounts. DNA hypermethylation may downregulate IFN-γ in AR cases,^109^ while DNA hypomethylation increases the mRNA amounts of IL-13 and IgE.^110^ Alterations in hypermethylation at CpG sites in the melatonin receptor 1 A gene may be caused by paternal genetic variations in AR.^111^

DNA methylation might also contribute to FA pathogenesis. Reports have shown differences in DNA methylation in some mitogen-activated protein kinase (MAPK) signaling genes, e.g., human leukocyte antigen (HLA)-DQB1 and the Treg-specific demethylation region (TSDR) of FOXP3. Differential genetic DNA methylation might also contribute to FA diagnosis.^39^ In a pilot study of cow’s milk protein (CMA, milk allergy), hypermethylation was found in the DEXH (Asp-Glu-X-His) box polypeptide 58 (Dhx58), zinc finger protein 81 (ZNF281) and HtrA serine peptidase 2 (HTRA2) regions.^112^ Maternal peanut allergy also induces epigenetic changes in the IL-4 promoter in the offspring, which is associated with Th2 immune response (production of IL-4 and IgE).^113^ In a study of identical monozygotic (MZ) twins, the distance between peanut allergy and nonallergy in methylation profiles containing 12 DNAm signatures was reduced compared with randomly paired individuals without genetic relationships, indicating peanut allergy-associated DNAm signatures might be linked to genetic factors.^114^

#### Histone modifications

The DNA is packaged into an organized chromatin structure formed by a core histone protein consisting of H2A, H2B, H3 and H4.^115^ Post-translational histone modifications mainly comprise acetylation, methylation, phosphorylation, ubiquitination, SUMOylation, and adenosine diphosphate (ADP) ribosylation of core histone tails, which reflect the epigenetic inheritance of many diseases, including AAS.^115^ Histone acetyltransferase (HAT)-mediated histone acetylation often loosens chromatin structure, facilitating access to transcription factors that induce gene expression. Conversely, histone deacetylation by histone deacetylases (HDACs) also leads to gene silencing. Higher levels of histone acetylation are generally associated with increased gene transcriptional activity and expression. Whether histone methylation is transcriptionally permissive or repressive depends largely on the number of methyl groups added and the position of the target amino acid residue in the histone tail.^34,116–118^

In adult asthmatic patients, lysine 18 acetylation of histone 3 (H3K18) and lysine 9 trimethylation of histone 3 (H3K9me3) are elevated in epithelial cells, and acetylation of H3K18 increases ΔNp63 (a p63 splice variant), epidermal growth factor receptor (EGFR) and signal transducer and activator of transcription 6 (STAT6) mRNA amounts.^119^ An imbalance of HAT and HDAC underlies impaired gene expression and is a determinant of asthma.^120^ HATs and HDACs have opposite functions, as the acetylation function of HATs promotes gene expression, while the deacetylation function of HDACs is responsible for gene silencing. In children with asthma, H3 acetylation of the FOXP3 gene contributes to Treg differentiation, and H3 histone acetylation critically affects the IL-13 gene promoter.^121^ Stefanowicz and collaborators assessed gene-specific alveolar epithelial histone acetylation and methylation statuses in asthma and healthy control cases, and found increased levels of H3K18ac and H3K9me3 in asthmatic patients.^119^ Acetylation of C-C Motif Chemokine 8 (CCL8), a neutrophil activator found in macrophages, as well as H3K18, results in elevated secreted amounts of this activator in airway smooth muscle.^122^ In asthma, the levels of CCR4 and CCL5 are high. CCR4 controls Th2 cell infiltration, while CCL5 is a leukocyte chemokine, and a single nucleotide polymorphism of CCR4 and CCL5 dimethylation (H3K4me2) is associated with Th2 differentiation.^123^ Resistance to steroid therapy in AAS has emerged, mainly due to IL-17A-induced steroid resistance resulting from decreased HDAC2 activity.^123^ Increased enrichment of transcriptionally active H3ac and H4ac histone markers found in AAS cases are associated with IL-13 upregulation in CD4^+^ T cells.^121^

In AR, increased HDAC activity may be involved in the pathogenetic mechanism by elevating pro-inflammatory cytokine amounts and reducing anti-inflammatory cytokine levels. Early responses are characterized by increased IL-4 expression,^124^ H3K9 acetylation, and H3K4 trimethylation at the IL4 locus.^125^ A study showed HDAC1 upregulation in nasal epithelial cells from AR patients,^126^ and IL-4 increased HDAC1 expression, leading to nasal epithelial barrier dysfunction. HDAC1 inhibition promotes the master regulators of T cell function, including IL-10 and CCL8, and prevents excessive activation of immune cells.^127^ Histone acetylation is also critical for AD pathogenesis. In AD pathogenesis, the demethylation, acetylation, and methylation of the H3 residue in the FOXP3 promoter gene region, along with the hypermethylation of the RORC gene and the methylation of the H3 residue, promote the regulation of Th0 cells. The differentiation of Tregs,^34,92,94,95^ thereby reduce the levels of histone acetylation at Th1 and regulatory sites.^128^

#### Non-coding RNAs in allergic diseases

Long noncoding RNAs (lncRNAs) are defined as transcripts longer than 200 nucleotides in length; functional RNAs that are not translated include micro-RNAs (miRNAs), small interfering RNAs (siRNAs), lncRNAs and Pivi-interacting RNAs (piRNAs). They are essential signaling and regulatory tools that affect transcriptional processes and may also alter gene expression post-transcriptionally, with critical roles in the development of allergic diseases. We take microRNAs as an example to explain their important roles in allergic diseases.

miRNA-21, high expression of miRNA155, and low levels of Let-7a were detected in peripheral blood specimens from asthmatic children. These markers can be used for the diagnosis and prognosis of childhood asthma;^129^ up-regulation of miR-126 in peripheral circulation is related to immune imbalance and is considered a biomarker for asthma diagnosis.^130^

MicroRNAs have critical functions in the development of allergic diseases. MiR-19b reduced airway remodeling and inflammation as well as oxidative stress by downregulating TSLP to inhibit Stat3 signaling in mice with experimental asthma.^131^ MMP-16 and ATG7 via miR-192-5p molecules reduce airway inflammation and remodeling.^132^ MiR-221 can control the enhanced airway smooth muscle cell proliferation in severe asthma cases.^133^ The circular RNA (circHIPK3) contributes to smooth muscle cell proliferation and airway remodeling in asthma patients via miR-326/ stromal interaction molecule 1 (STIM1) signaling.^134^ MiR-130a-3p and miR-142-5p mediate lung macrophage polarization and are associated with airway remodeling.^135^ MiR-155 and miR-221 are closely associated with the regulation of Th2 responses and airway smooth muscle hyperproliferation in asthmatic patients.^133,136^ Vascular endothelial growth factor A (VEGF-A) amounts are elevated in sputum and serum samples from asthmatics, and has-miR-15a is associated with VEGF-A downregulation in CD4^+^ T cells.^137^ MiR-21 downregulates IL-3, IL-5 and IL-12, and inhibits IFN-γ and IL-12 production by dendritic cells, and reduces IFN-γ biosynthesis in CD4^+^ T cells.^138^ MiR-21 overexpression was also associated with the differentiation of Th2 cells in vitro. In granulocyte-infiltrating asthma, miR-221-3p in epithelial cells and sputum was inversely associated with airway eosinophilia.^139^ Downregulated miR-28-5p and miR-146a/b activate blood CD8^+^ T cells in severe asthma.^140^ MiR-223-3p, miR-142-3p, and miR-629-3p are involved in severe neutrophilic cellular asthma.^141^ MiR-126 induces Th2-type eosinophilic asthma,^142^ and miR-23-27-24 regulates T cell function and differentiation; meanwhile, miR-24 and miR-27 suppress Th2 cell differentiation, leading to IL-4 cytokines.^143^ Recently published reports revealed miR-200a is involved in asthma pathogenesis via phosphatidylinositol 3 kinase (PI3K)/ RAC-alpha serine/threonine-protein kinase (AKT) signaling.^144–146^ In childhood asthma, the miR-29c/B7-H3 axis controls the differentiation of Th2/Th17 cells, and the above microRNA studies might point to novel research directions for developing treatments for AAS.^147^

In AR, aberrantly expressed circulating lnc-NEAT1 and miR-125a were associated with Th2 cell percentage and symptoms in pediatric AR.^148^ In AD, Liew et al. found decreased expression of miR-335 in AD lesions compared with healthy control skin.^149^ Nuclear factor kappa-B (NF-κB) (p65) is a critical modulator of inflammatory immune response, and miR-124 is associated with inflammatory response and may constitute a new effector and regulator of NF-κB.^150,151^ In diseased skin, miR-124 is downregulated in AD patients.^152^ In macrophages, miR-155 targets IL-13Rα1.^153^ In dendritic cells (DCs), miR-221 knockdown or miR-155 overexpression promotes apoptosis, while miR-155 overexpression in mDCs enhances the production of IL-12p70.^154^ The study of microRNA would bring new hope in the treatment and understanding of allergic diseases.

### Cell signaling pathways play critical roles in allergic diseases

Allergic diseases are immune disorders caused by an imbalance of the immunity, in which immune cells play an important role. Signaling pathways are important in intercellular signaling. This review provides a systematic review of the signaling pathways involved in allergic diseases from the nucleus to the cell membrane, in the hope of laying a solid foundation for the study of allergic diseases.

#### Notch signaling pathway

In the 1910s, the Notch gene was detected in *Drosophila melanogaster* with notched wings.^155,156^ Notch signaling is highly conserved. In mammals, NOTCH has four paralogs, including NOTCH1-4, with redundancy and distinct roles.^157^ Human Notch1-4 genes map to chromosomes 9, 1, 19 and 6, respectively. The NOTCH receptor undergoes three cleavages and is transferred to the nuclear compartment to regulate target genes transcriptionally. Notch signaling is divided into the canonical and non-canonical pathways with complex functions, but the pathway is now well known.^158^ It is mainly involved in diverse molecular events across species, including tissue functional damage and repair; abnormal Notch pathway might lead to different pathological processes.

**Fig. 3:**
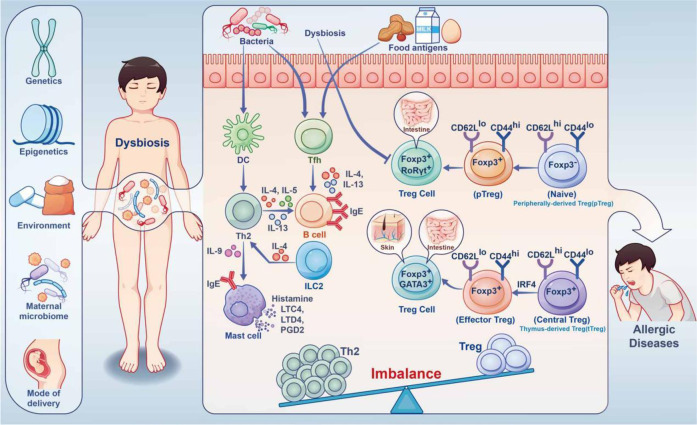
Immune imbalance caused by dysbiosis under the combined effect of gene environment in IgE-related FA. During childhood, the human microbiota is influenced by a combination of the maternal microbiome, mode of delivery, genetics, epigenetics, environment, etc. Dysbiosis resulting from aberrant damage to the gut microbiota early in life impairs Treg differentiation. This results in imbalance of Treg and Th2 cells. Food allergens and the microbiota promote T follicular helper (Tfh) responses to induce B cells, which produce large amounts of IgE through IL-4, IL-13 cytokines, causing allergic reactions

In AAS, eosinophilic asthma is dominated by Th2-type immune responses, and Notch signaling upregulates the key transcription factor Gata3.^159,160^ NOTCH4 is known to be critical for asthma development (Fig. 3). Repeated allergen exposure induces Tregs that produce high amounts of Notch4, which activates downstream Wnt and Hippo pathways, thereby promoting the transformation of iTregs into Th2 and Th17 cells, and exacerbating AAS.^160,161^

In other allergic diseases, such as AR, FA, and AD, the exact underpinning mechanism remains unclear, Notch signaling also plays important roles. In a study of AR, the serum amounts of Notch1 and Jagged1 (Jag1) in AR patients were significantly increased, which was confirmed in mouse experiments. In this study, Notch signaling could downregulate Foxp3 expression and inhibit Treg differentiation, thereby promoting AR occurrence and development.^162^ In a FA study, blocking the Notch signaling pathway could suppress Th2 polarization, increase Th1 cell differentiation and promote Th1/Th2 balance in a mouse model, thereby preliminarily verifying that blocking the Notch signaling pathway inhibits ovalbumin (OVA)-induced FA.^163^ In addition, Tfh cell production and function are dependent on Notch signaling, Notch receptors 1 and 2 are required for Tfh cell production, and Notch signaling enhances the production of type 2 cytokines in Tfh cells.^164^ Administration of a Notch signaling inhibitor inhibits IgE-mediated proliferation of intestinal mucosal mast cells (MMCs) in mice with food hypersensitivity, thereby attenuating allergic diarrhea and anaphylaxis.^165^ In AD, the epidermis of AD patients exhibits a marked deficiency in Notch receptors, leading to the upregulation of alarm TSLP, which triggers Th2-associated responses as well as TSLP and IL-31-related pruritus.^166,167^ Keratinocyte-produced TSLP and granulocyte-macrophage colony stimulating factor (GM-CSF) induce macrophage and DC activation, drive Th2 polarization, and promote eosinophil and mast cell infiltration, thereby enhancing the immune response.^168,169^

#### JAK/STAT signaling pathway

Janus kinase/signal transducer and activator of transcription (JAK/STAT) signaling represents a relatively simple membrane-nucleus pathway that mainly upregulates diverse key modulators involved in cancer and inflammatory processes. The well-conserved JAK/STAT pathway consists of ligand-receptor complexes, JAK and STAT. JAK consists of four cytoplasmic tyrosine kinases, i.e., JAK1, JAK2, JAK3 and TYK2. STAT proteins comprise STAT1, STAT2, STAT3, STAT4, STAT5a, STAT5b and STAT6. Each cytokine requires interaction with specific receptors on its target cells to activate this pathway, and these receptors contain associated intracellular domains composed of JAK family members.^170^ JAKs are inactive before exposure to cytokines, which induce JAK activation through phosphorylation by binding to their receptors.^171–173^ Activated JAKs phosphorylate the receptors on specific tyrosine moieties in the intracellular tail.^174^ STATs at receptor sites are also phosphorylated by JAKs.^175,176^ STATs are phosphorylated and translocated into the nuclear compartment to transcriptionally upregulate specific genes,^177^ often leading to cell proliferation or differentiation.

JAK/STAT signaling is highly involved in the differentiation of Th cell subsets; Th1 cell differentiation is controlled by the IFN-γ/STAT1 and IL-12/STAT4 signaling pathways.^178,179^ Meanwhile, Th2 cell differentiation is modulated by IL-2/STAT5 and IL-4/STAT6 signaling.^180,181^ The differentiation of Th17 cells mainly requires the involvement of STAT3/STAT4 signaling induced by IL-6 or IL-23.^182^ In allergic diseases, the JAK/STAT pathway is critical for cell proliferation and differentiation. In both rat and human bronchial smooth muscle cells, IL-13 induces JAK1-STAT6 signaling, which regulates Ras Homolog Family Member A (RhoA) activation that promotes smooth muscle contraction.^183,184^ IL-4 and IL-13 induce STAT6 in target cells with JAK involvement, and gene-targeted knockout mouse assays revealed STAT6 contributes to IgE synthesis, bronchial hyperresponsiveness and airway remodeling upon allergen sensitization.^185,186^ Further type 2 asthma-related cytokines, including IL-5 and TSLP, signal through JAK-dependent pathways. The JAK signaling pathway is critical for the differentiation of naive precursors into CD4^+^ Th2 cells, and the key cytokines involved are IL-2 and IL-4, which bind to cytokine receptors coupled to JAK1 and JAK3, respectively, then induce STAT5 and STAT6.^187^

In AAS, cytokine receptors, e.g., IL-4, IL-5, IL-13, IL-31, and TSLP, promote JAK/STAT signaling activation.^188,189^ In AD, Th2 immune enhancement induced by JAK/STAT signaling downstream of multiple cytokines, including IL-4, IL-5 and IL-13, is considered an essential pathogenic pathway.^190^ It was demonstrated that the JAK-STAT pathway regulates inflammatory processes and induces changes in the natural skin barrier, increasing TEWL (transepidermal water loss) by upregulating IFN-γ, IL-31, IL-23, and IL-22.^190^ STAT3 is one of the factors responsible for IL-23 expression induced by IL-6 from DCs, which is critical for Th17 lymphocyte differentiation and cellular memory, leading to a disruption of epithelial barrier integration.^191^

#### NF-κB/MAPK signaling pathway

By 2022, NF-κB has been known for 36 years. NF-κB (nuclear factor) is a protein factor with gene transcriptional regulation, which is present in almost all nucleated cells. When cells are stimulated by inflammatory mediators, NF-κB protein is activated in the cytoplasm and enters the nucleus to regulate the expression of various inflammatory factors, playing an important role in allergic diseases.

**Fig. 4:**
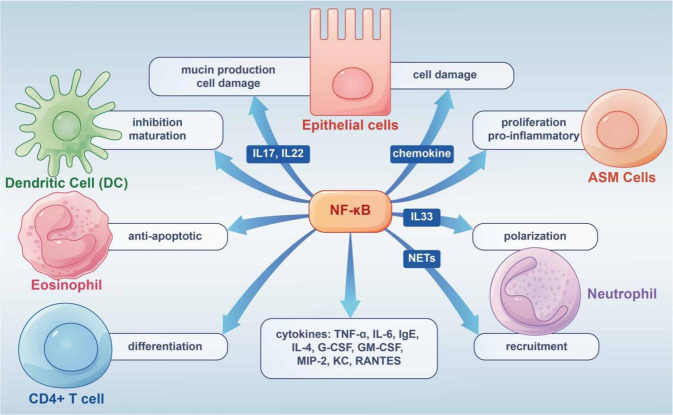
Graphical summary of NF-κB pathway’s role in allergic diseases. The NF-κB pathway is highly involved in the occurrence and development of allergic diseases by acting on different cells and releasing inflammatory factors

In AAS, the NF-κB/MAPK pathway controls inflammatory and immune responses by regulating TNF-α and IL-6.^192–194^ A 2019 study demonstrated that after nuclear translocation of phosphorylated P65, inhibited NF-κB/MAPK signaling may modulate IgE and IL-4 production.^195^ Enhanced NF-κB nuclear binding or production was also found in inflammatory cells collected from induced sputum in asthmatic patients.^196^ Besides, experiments have shown enhanced NF-κB activation in airway tissues and inflammatory cells challenged by allergens such as ovalbumin (OVA) and house dust mite (HDM) extract.^197–199^ In addition, the studies related to increased Tfh cells in allergic diseases, NF-κB deficiency led to a decrease in CXCR5 (Tfh cells expressing chemokine receptors) in mice and a consequent decrease in the number of Tfh cells.^200^ All these studies suggest that NF-κB signaling is essential in the cellular immune response of allergic diseases, especially AAS. The following focuses on the functions of NF-κB in immune cells in allergic diseases (Fig. 4).

##### Epithelial Cells

Epithelial cells play a key role in airway diseases and constitute a critical interface between the body and the environment.^201^ The epithelium coordinates responses to diverse invasive injuries via the production of multiple immunomodulators and inflammatory factors controlled by NF-κB. The latter mediators mostly comprise chemoattractants that induce inflammatory cell infiltration affecting epithelial function; among these, TSLP synthesized by bronchial epithelial cells is essential in Th2 responses that trigger allergic airway inflammation.^201^ The neutrophil-associated proteins S100A8 and S100A9 induce mucin production in airway epithelial cells through toll-like receptor 4 (TLR4)-related NF-κB pathway activation during AAS attacks.^202^

In isolated mouse airway epithelial cells, NF-kB activation upregulates IL-6, granulocyte colony-stimulating factor (G-CSF), GM-CSF, macrophage inflammatory Protein 2 (MIP-2), keratinocyte-derived chemokine (KC), and RANTES. NF-κB activation in mouse epithelial cells also leads to the recruitment of neutrophils for innate immune response.^203^ In the OVA-induced model of allergic diseases, NF-κB activation leads to airway inflammation, goblet cell hyperplasia, and induced expression inflammatory cytokines, including IL-15, IL-10, and IL-9.^204^

##### Airway Smooth Muscle (ASM) Cells, Neutrophils and Eosinophils

In AAS, acute contraction of ASM is the main factor that causes bronchospasm. ASM cells contribute to persistent histological alterations in the airway wall, ASM and inflammatory cells are important players in inflammation,^205^ in which thrombin and IL-1α stimulate NF-κB signaling in ASM cells.^206^ IL-8 over-secretion by ASM cells may increase NF-κB’s binding to the IL-8 promoter.^207^

AAS includes eosinophilic, neutrophilic, oligogranulocytic and mixed granulocytic types, based on the type of inflammatory infiltrating cells. Of these, neutrophil infiltration is an important cause of AAS exacerbation and resistance to hormone therapy.^208^ Th17 lymphocytes are critical for neutrophilic asthma, and are the major producers of IL-17A, IL-17F and IL-22, whose amounts are elevated in the airways of severe steroid-refractory asthma cases.^209^ NF-κB signaling is associated with IL-17 and/or IL-22-related production of epithelial mucin and ASM cell proliferation.^210–212^ IL-33 promotes neutrophil polarization via c-Jun N-terminal kinase and NF-κB-related pathways. NETs induce CXCL1, CXCL2, and CXCL8 expression in airway cells through TLR4/NF-κB signaling, thereby recruiting neutrophils to inflammatory sites.^213^ In neutrophilic asthma (NA) mice, NETs trigger the expression of chemokines by airway and alveolar epithelial cells that promote the recruitment of more neutrophils through the TLR4/NF-κB pathway, leading to epithelial cell damage.^214^

Airway inflammation in eosinophilic allergic asthma features infiltrated and activated eosinophils, and co-culture of epithelial cells with mast cells or eosinophils induce NF-κB-dependent cytokine production by airway epithelial cells.^215,216^ NF-κB signaling is critical in the survival of eosinophils, exerting an anti-apoptotic effect through autocrine TNF-α.^217^ NF-κB suppressors on the other hand, including MG-132, reduce eosinophil amounts and alleviate allergic inflammation.^218^

##### Dendritic cells and Lymphocytes

DCs interconnect innate and adaptive immune systems, with crucial roles in promoting immune defense and maintaining immune tolerance. Previous reports have established NF-κB signaling involvement in DC development, with NF-κB suppression preventing DC maturation associated with the upregulation of MHC and co-stimulatory molecules.^219^

In eosinophilic allergic asthma, naive T cells differentiate and mature into Th2 cells, which biosynthesize IL-4, IL-5 and IL-13 with NF-κB involvement, and stimulate B lymphocytes to produce immunoglobulin E (IgE).^220^ During CD4^+^ T cell differentiation, IL-6 and TGF-β are highly involved in Th17 cell differentiation.^221^ NF-κB signaling regulates antigen-presenting cell function and controls CD4^+^ T cell differentiation into Th effector cells.^222,223^ However, the present study is still inconclusive.

#### Hippo signaling and allergic disease

Hippo signaling was first discovered in *Drosophila* and is a highly conserved pathway.^224^ It mainly comprises the cascade kinase cascade transcription molecule mammalian STE20-like kinase 1/2 (MST1/2), WW domain of Sav family containing protein 1 (SAV1), and MOB kinase activator 1 (MOB1), with large tumor suppressor 1/2 (LATS1/2) upstream and Yes-associated protein (YAP)/effector molecules with PDZ- binding motif (TAZ) downstream. When Hippo signaling is not activated, unphosphorylated YAP undergoes nuclear translocation and interacts with TEAD, thereby triggering the transcription of target genes. After Hippo signaling activation, TAOK induces MST1/2 phosphorylation, and phosphorylated MST1/2 interacts with SAV1 for MST1/2-SAV1 complex formation. With activated MOB1, the latter complex phosphorylates LATS1/2. In turn, LATS1/2 phosphorylation triggers YAP activation, causing YAP capture by 4-3-3 proteins in the cytosol or degradation by SCFβ-TRCP E3 ubiquitin ligase-mediated ubiquitin-proteasome signaling.^225^

**Fig. 5:**
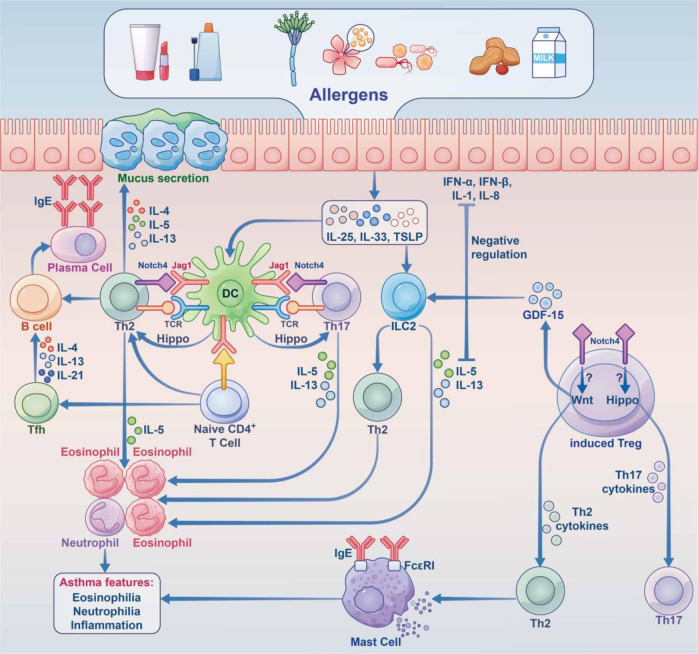
The roles of the hippo and Notch pathways in AAS. Under stimulation by allergens, epithelial cells synthesize large amounts of proinflammatory cytokines (IL-25, IL-33, TSLP, etc.), thereby acting on innate lymphocytes (ILC2 cells) and DCs. Jag1 on DCs interacts with Notch receptors on T cells for Notch pathway induction. Notch transforms induced Tregs into Th2 and Th17 cells. Naive CD4 + cells affect Tfh cell class switch recombination by secreting IL-5, thus acting on B cells to induce plasma cells, which produce IgE. At the same time, Th2 cells secrete IL-4 and others to activate B cells to synthesize IgE, which interacts with IgE receptors on mast cells. In case the allergen invades the body again, it directly cross-links with IgE on the cell surface and releases a variety of active mediators, which trigger the clinical symptoms of asthma. Th17 cells are activated through the Hippo pathway; Th2 cells are activated through the Wnt pathway, and GDF-15 molecules are stimulated to act on ILC2 cells to enhance the expression of IL-13, although this remains controversial. Notch converts induced Tregs into Th2 and Th17 cells via hippo pathway-dependent mechanisms. IL interleukin, TSLP thymic stromal lymphopoietin, ILC2 group 2 innate lymphoid cell, DC dendritic cell, Jag1 jagged1

In AAS, Hippo signaling mainly induces cell differentiation. The Notch4 protein mediates immune tolerance and leads to Treg dysfunction, thereby promoting allergic airway inflammation.^226^ After alveolar macrophage engulfment of allergens and particulate pollutants, Jag1 is highly expressed on alveolar macrophages, thereby activating Notch on CD4^+^ T cells and promoting inflammation associated with Th2 and Th17 effector T (Teff) cells;^227,228^ at the same time, alveolar macrophages secrete a large amount of IL-6, which promote the expression of Notch4 on induced regulatory T (iTreg) cells, thereby activating Hippo signaling, which further exacerbates Th17 cell-induced inflammation (Fig. 5).^161^ In other allergic diseases, including AR, AD and FA, no associations with Hippo signaling have been reported.

The newly discovered Hippo pathway plays a critical role in the immune function of the body, with complex crosstalk with other signaling pathways, and is regulated by other signaling pathways (such as Notch, Wnt signaling pathway, etc.). As the study of Hippo signaling pathway continues to deepen, its important function in allergic diseases will be gradually discovered.

#### TOLL-like receptor (TLR) signaling pathway

The increasing prevalence of allergic diseases is not only related to changes in the modern living environment (such as pollution, low-endotoxin living environments, smoking, etc.), which may induce the disorder of immune system,^229,230^ but also closely related to the loss of microbial biodiversity.

TLRs represents an important group of transmembrane protein receptors that are critical for proper activation of innate immunity, are highly conserved, and comprise binding domains containing arginine-rich repeats. As molecules involved in the first line of defense, TLRs are induced by pathogen-associated molecular patterns (PAMPs), which are found in diverse pathogenic organisms and absent from the host. There are eleven known human TLRs (TLR1 to TLR11), all with functions except for TLR11.^231,232^ TLRs are located on and within immune and non-immune cells, respectively, and are critical for initiating adaptive immune responses, including in alveolar macrophages, mast cells, epithelial cells, neutrophils, natural killer cells, and antigen presenting cells (APCs). Different TLRs recognize various groups of molecules in diverse pathogens. For example, multiple diacyl peptides can be recognized by TLR1 and TLR6, while liposomes are recognized by TLR1/2.^233^ TLR5 cooperates with TLR4 to recognize bacterial flagellin.^234^ Studies have shown that gut microbiota regulates the activity of Th1 and Th2, thereby affecting the formation of immune tolerance in the body.^235^ To maintain immune homeostasis, the activation of innate immune cells requires TLR signaling molecules, and innate immunity should be activated correctly to stimulate the body’s immune response.^231^

The strength of the TLR signaling pathway determines the possibility of allergic diseases. Accumulation of TLR4 signaling on DC was detected in house dust extract (HDE) hypersensitivity.^236,237^ Bacteria belonging to the healthy lung microbiome elicit baseline TLR response, whereas those involved in asthma show stronger TLR response. Mice administered asthma-associated proteobacteria show elevated amounts of neutrophils and cytokines in comparison with animals administered commensal *Provetella*.^238^

The strength of TLR signaling does determine the occurrence of allergic reactions or its absence. Strong TLR signals are protective against allergic airway disease, while low airway amounts of TLR ligands cause airway sensitization and Th2-type immunity.^239–241^ Studies have shown that low-level flagellin (TLR5 ligand) can enhance OVA-associated hypersensitivity in mice, whereas a high flagellin dose protects the animals from hypersensitivity by producing CD25^+^ Treg-dependent regulatory DCs and T cells.^242^ Differential responses to TLR activation have been considered a shift from Th2-type immunity to Th1-type immunity with increasing stimulation intensity.

In AAS, alveolar macrophages are immune cells with critical roles in the clearance of immune antigens. Excessive inflammatory response of these cells, however, might induce tissue damage.^243^ Alveolar macrophages are important in developing tolerance to inhaled allergens by inhibiting T cell proliferation and APC function.^244^ Asthmatic patients have reduced monocyte and macrophage amounts in comparison with healthy control individuals.^245^ TLR2, 4, 5, 6, 7, 8 and 9 are expressed on macrophages. TLRs have a critical function in AAS; among the TLRs expressed by human lung cells, TLR1-5, 7 and 8 have the highest expression.^246^ TLR2-6 and TLR9 are highly expressed on human airway epithelial cells.^247,248^ Mouse alveolar macrophages highly express TLR2, 4 and 9,^249^ and mouse macrophages produce TLR1-7 and 9 mRNAs.^250,251^

Lipopeptides in Gram-positive bacterial organisms and mycoplasmas show diacylation, while lipopeptides in Gram-negative bacterial and mycobacterial species show triacylation. When TLR2 polymerizes with TLR1 or TLR6, the lipopeptide is recognized by TLR2. The TLR2/TLR6 heterodimer recognizes diacylated lipopeptides such as S-FSL1 (TLR2/6), R-FSL1 (TLR2/6/CD36) and MALP-2 (macrophage-activating lipopeptide-2). Cell wall constituents in Gram-positive bacteria, including lipoteichoic acid, also bind to the TLR2/TLR6 heterodimer.^252^ Phagocytic cells, airway epithelial cells, smooth muscle cells, glia, mouse bone marrow-derived mast cells, and B cells all can express the TLR2 receptor.^253^ In OVA-sensitized animal model, TLR2 receptor pathway induction also results in enhanced pause and increased bronchioalveolar lavage fluid (BALF) eosinophil amounts.^254,255^ Elevated TLR2 ligand amounts can also elevate serum IgE concentration. Though TLR6 is heterodimerized with TLR2, and the heterodimer plays an important role in AAS, TLR6 is decreased in PBMC compared with healthy controls, and is also overexpressed in severe asthma cases compared with mild asthmatic patients.^256^ TLR4 uses the adaptor protein TRIF via myeloid differentiation primary response gene 88 (MyD88)-dependent and MyD88-independent pathway, respectively, enhancing IRF-3 induction and IFN-β production.^257^ In bronchial asthma cases, it was shown that TLR4 activation of macrophages produces cytokines that affects immune balance and thus affects the Th1/Th2 balance.^258^ After TLR5 recognizes flagellin, it induces NF-κB signaling through MyD88 and TNF receptor associated factor 6 (TRAF6), producing cytokines to trigger an inflammatory response.^245^ Recently, Nawijn et al. showed that intranasal TLR2 induction by aerosolized allergens promotes allergen-specific Treg proliferation to suppress asthma in a mouse model.^259^ Numerous studies have shown that TLR2 stimulation by parenteral or mucosal treatment with synthetic agonists prevents APCs from triggering Th2-polarizing responses, reducing IgE antibodies and immunogenicity in a mouse model of asthma.^260–263^

In FA, Treg activation is an important pathway by which gut DCs and macrophages induce immune tolerance, disrupting the normal immune homeostasis of the intestine.^264^ All microbial pattern recognition receptors (PRRs) may be involved in food tolerance and allergen presentation. TLR2 is highly expressed by intestinal epithelial cells (IECs) and DCs, and most commensal bacteria in the intestine are gram-positive organisms, meaning they have a high ability to induce TLR2.^265,266^ In AR, researchers were surprised to find that TLR4 inhibits allergic response in OVA-induced AR in a mouse model.^267^ TLR2, 3 and 4 were highly expressed in nasal mucosa specimens from AR cases in a study of 27 healthy control individuals and 42 cases of seasonal allergic rhinitis.^268^ All these studies have once again confirmed that TLR is critical for the etiology and progression of allergic disorders, which is worthy of further exploration.

#### Wnt/β-catenin signaling

In 1982, the Wnt gene was described as integrase-1 in murine breast cancer cells and the wingless gene in Drosophila.^269^ Wnt signaling plays core roles in the maintenance of progenitor cells and stem cells, the differentiation of T cells, and the regulation of cellular immunity. Among the Wnt-mediated signaling pathways, the most classical Wnt/β-catenin pathway contributes to maintaining human tissue homeostasis. Wnt, which mediates extracellular signals, is a secretory glycoprotein, with 19 human Wnt proteins reported as of now. This pathway relies on β-catenin, which is activated by binding extracellular Wnt ligands to membrane receptors through autocrine and paracrine processes, inhibiting the degradation of β-catenin so that it can be stably accumulated in the cytoplasm and transferred to the nuclear compartment, where it can work together with T cell factor/lymphoenhancer binding factor to stimulate the transcription of target genes.^270,271^

Wnt/β-catenin contributes to airway remodeling in asthma by upregulating the tenascin C/platelet-derived growth factor receptor (PDGFR) or activating p38 MAPK and its target genes c-Myc and cyclin D1 to induce proliferation in airway smooth muscle cells.^272,273^ A study by Trischler et al. revealed that Wnt10b, known as the classical Wnt ligand, is highly produced by T cells in AAS, and its absence increases the activation of cultured T cells and enhances immune response in animal models.^274^ In addition, after mesenchymal stem cell-derived exosomes and vitamin D inhibit Wnt/β-catenin signaling, airway remodeling is reduced, thereby inhibiting chronic allergic inflammation in the airway.^275,276^ Upregulated Notch4 in blood Tregs from asthma patients differentiates Tregs into Th2 and Th17 T cells through a Wnt and Hippo pathway-dependent mechanism, and Wnt induction upregulates growth and differentiation factor 15 (GDF15) in Tregs, and this feedforward mechanism exacerbates inflammation.^226^ However, Wnt-1/β-catenin pathway induction promotes allergic airway diseases. Overexpression of Wnt1 reduces DC migration to draining lymph nodes and induces an appropriate T cell tolerance response without causing T cell proliferation.^277^

Related literature reported that inactivation of Wnt/β-catenin signaling could reduce nasal mucosa damage and eosinophil infiltration, decrease the infiltration of nasal mast cells and enhance red blood cell immune adhesion, thereby reducing the progression of AR.^278^

There are few reports on this pathway in FA and AR. Research in allergic dermatitis shows that Notch deficiency is the basis for inhibiting epidermal differentiation and skin barrier defects, thus enhancing Wnt pathway, which is very important for the proliferation of epidermis cells.^279^

#### PI3K/AKT signaling

PI3K in the lipid kinase family interacts with the PH domain of the AKT protein (also known as PKB), inducing its conformational change and AKT phosphorylation. Activated AKT is transferred from the cytosol to the plasma membrane, and subsequently induces its downstream effectors, including mammalian target of rapamycin (mTOR).^280^

PI3K inhibitors have been considered to have great potential in the treatment of inflammation. Current evidence shows that PI3K participates in the pathogenesis of asthma through two main mechanisms. First, PI3K increases the permeability of mouse blood vessels to enhance antigen-induced airway inflammation and high reactivity.^281–283^ In the OVA-induced asthma model, inhibition of PI3K110δ subtype (PI3K-δ) reduces the activation of HIF-1α in airway epithelial cells, as well as antigen-induced airway inflammatory reactions and hyperresponsiveness, by regulating vascular leakage mediated by VEGF.^284^ Secondly, the PI3K pathway induces airway smooth muscle cell proliferation, promotes airway smooth muscle thickening and luminal stenosis, thereby participating in airway remodeling.^285^ With respect to immune cells, PI3K regulates the differentiation of Th cells and eosinophils in asthma,^286,287^ and affects the occurrence and development of inflammation in asthma. In an animal model of AAS, it was found that PI3K and AKT activities in lung tissue are increased, as well as the expression of mTOR. After treatment with a PI3K inhibitor, some pathological manifestations of asthma (such as increased amounts of activated chemokines in eosinophils, bronchoalveolar lavage fluid IL-5 and IL-13, lung tissue eosinophilia, increased mucus secretion in the respiratory tract, airway hyperresponsiveness, etc.) are obviously inhibited, and PI3K/AKT signaling highly regulates asthma pathogenesis.^288^

In AR, drug development research found that the anti-allergic drug α-TCP (alpha-tocopherol) and the androgen receptor antagonist bicalutamide both play anti-inflammatory and alleviating roles in AR in animal models by inhibiting the PI3K/AKT/mTOR pathway in mast cells.^289,290^ A mouse model with AR shows that increased leptin enhances the expression of type II innate lymphoid cell (ILC2) transcription factor and type II cytokines through the PI3K/AKT pathway.^291^ For mice lacking CCR3 gene in the bone marrow, the activity of PI3K/AKT signaling was also significantly reduced, and nasal eosinophil infiltration was inhibited; in addition, serum Th2 cytokines were reduced, and the symptoms of AR in mice were alleviated.^292^ It was found in cell experiments that ST2/PI3K/mTOR-mediated autophagy is inhibited by IL-33 secreted by nasal epithelial cells, thereby promoting mast cell degranulation in allergic asthma.^293^

In the skin, dysregulated PI3K/AKT pathway might result in serious pathologies featuring unchecked cell proliferation and inflammatory response.^294^ In addition, PI3K/AKT signaling also modulates mast cell degranulation via miRNAs in allergic skin diseases. High-expression miR-126 induces IgE-mediated mast cell degranulation related to PI3K/AKT signaling by increasing Ca^2+^ influx.^295^

Serum chitinase 3-like 1 (CHI3L1) amounts are increased in individuals with allergic disorders and promote Th2-related immunity and the polarization of M2 macrophages through PI3K/AKT signaling in FA.^296^

#### mTOR signaling pathway

mTOR represents a serine/threonine protein kinase that belongs to the PI3K-associated protein kinase (PIKK) family.^297^ While associated with the above paths, mTOR signaling may serve as an upstream response to other signaling pathways. mTOR has catalytic subunits in two different complexes, including mTOR complex 1 (mTORC1) and mTORC2,^298^ which have different susceptibilities to rapamycin, substrates, and functions.^299^ Studies in allergic diseases have shown that mTOR is a key molecule for sensing the immune microenvironment and determining the function and differentiation of immune cells,^300^ because it regulates a variety of immune cells and limits pro-inflammatory mediators.^301,302^ For example, increasing evidence supports that mTOR is an important regulator of Tfh cell differentiation. The balance of Tfh and Th1 cell differentiation in vivo is regulated by IL-2 signaling through PI3K, AKT and mTOR, and both mTORC1 and mTORC2 essentially promote Tfh cell differentiation and germinal centers (GC) formation, which cannot be ignored in allergic diseases.^303,304^

In AAS, the progenitor cells of granulocytes originate from the bone marrow and move to blood vessels and lungs when inflammation occurs. Among them, eosinophils regulate Th2 immune response, which is related to the severity of the disease,^305^ while ablation of mTOR leads to Gata-1 overexpression and increases eosinophil differentiation.^306^ The angiogenic factor fibroblast growth factor-binding protein 1 (FGFBP1) is highly expressed in asthma models with airway remodeling features, because activating mTORC1 and signal transducer and activator of transcription 3 (STAT3) signaling pathways enhances FGFBP1 expression and secretion, thus inducing angiogenesis.^307^

The target protein mTOR of rapamycin is involved in the growth of keratinocytes. Studies have found IL-13 activates the mTOR signaling pathway and downregulates miR-143, followed by the downregulation of epidermal barrier related proteins. Therefore, rapamycin could treat allergic dermatitis by inhibiting mTOR.^308^

#### FcƐRI signaling pathway

Fc receptors play major roles in adaptive immunity by interacting with immunoglobulins, among which FcεRI represents a high-affinity IgE receptor found on mast cells, basophils, eosinophils and APCs.^309^ When IgE binds to FcεRI to trigger immunity, FcεRI aggregation induces a variety of signaling pathways to regulate the secretion of allergy-associated mediators, including histamines and leukotrienes, and induces the transcription of Th2 cytokines and tumor necrosis factor (TNF) genes,^310^ which leads to potentially life-threatening allergic diseases. The tetrameric form of FcεRI is present in mast cells and basophils, while the trimeric form is found in other immune cells; FcεRIα, FcεRIβ and FcεRIγ (αβγ2) are encoded by the FcεR1A, FcεR1B (MS4A2) and FcεR1G genes, respectively.^311,312^

The pro-inflammatory effects mediated by FcεRI in different allergic disorders have the following similar mechanisms. Signal transduction in mast cells is induced by the phosphorylation of immune receptor tyrosine activation motif (ITAM) of FcεRIβ and FcεRIγ subunits by Src-protein tyrosine kinase. This results in the recruitment of tyrosine kinase Syk, which mediates the activation of some adaptor molecules (SLP76, LAT, etc.), leading to calcium mobilization.^313^ Therefore, dephosphorylation of tyrosine kinase activating signals downstream of the IgE-FcεRI complex may prevent allergic diseases,^314^ and TLR-mediated release of cytokines from mast cells depends on the expansion effect of FcεRI, which is more important in the late reactions associated with inflammation.^315^ There is a synergistic effect between TLR and FcεRI-mediated activation in basophils, which promotes Th2 cell differentiation and induces degranulation and cytokine release.^316,317^ Platelets depend on the interaction between allergens and allergen-specific IgE and FcεRI, and are directly involved in allergic asthma.^318^ In addition, FcεRI signaling can also activate the PI3K signaling pathway.^314^

In allergic diseases, sensory neurons exposed to allergens produce action potentials; the Ca^2+^ flux mediated by immune complexes increases, the action potentials discharge and neuropeptides are released, thereby causing pain or itching. The IgE receptor FcɛRI highly contributes to the development and remodeling of airway inflammation in allergic asthma.^319^ Studies in animals with experimental allergic asthma demonstrated that when the vagus nerve, which dominates the airway, senses the invasion of allergens, pain receptor neurons overexpress the immunoglobulin receptor FcɛRI and release Substance P, which drives the polarization of Th2 cells, thus triggering allergic inflammation.^320^

IgE-mediated FA is very common, and FcɛRI is also upregulated in the abdominal vagus nerve of mice with experimental food allergy, which promotes the skewed Th2 polarization in the intestine.^321^ Functional FcɛRI also exists in intestinal neurons, and stimulation of IgE antigen activates intermuscular neurons.^322^

#### NOD-like receptors signaling pathway

Nucleotide-binding oligomerization domain (NOD)-like receptors (NLRs) are located in the cytoplasm, and 23 species have been found in the human body to date.^323,324^ This pattern recognition receptor recognizes microbial compounds such as PAMPs and Damage-Associated Molecular Patterns (DAMPs) and cooperate with TLR and related pathways to trigger antibacterial immune response. The NOD-like receptor consists of the following three domains: (i) the nucleotide sequence located in the center has a domain that binds to NACHT, which drives the activation of downstream inflammatory caspase and NF-κB; (ii) the effector domain located at the N-terminal end mediates the interaction with adaptor proteins and downstream effectors and transmitting receptor excitability information; (iii) the C-terminal region is composed of leucine-rich repeats (LRRs), which constitute the microbial pattern recognition domain.^325,326^ NLRs recognize many pathogen-related model molecules, including microorganisms and toxins secreted by microorganisms^327^ which could be used for immune surveillance and host defense. The NLR signaling pathway mainly has the following functions: signal transduction, inflammasome formation, gene transcription stimulation and autophagy.^328,329^

After an NLR recognizes bacteria-related ligands, γ-D-glutamyl-meso- diaminopimelic acid (iE-DAP) and muramyl dipeptides (MDP), it promotes the expression of the adhesion molecule ICAM-1 on eosinophils and bronchial epithelial cells, and thus drive the cell adhesion, chemotaxis and migration of leukocytes. NLRs also induce eosinophils and bronchial epithelial cells to produce pro-inflammatory molecules such as IL-1β, IL-6, CXCL8, CCL2, CCL3, CCL4, and CCL5, thereby causing lung inflammation.^330^

In animal models, treatment of allergic asthma mice with NOD1 ligand induces subcutaneous fibrosis and significantly increases serum amounts of total IgE, eosinophils and the chemokine CCL5 as well as bronchoalveolar lavage fluid amounts of the Th2 cytokine IL-13.^331^ However, intranasal NOD2 ligand induces the expression of TSLP, IL-25 and OX40L in the lung,^332^ and these three molecules have been reported to promote asthma-related inflammation,^333–335^ blunt the production of antigen-specific CD4^+^Foxp3^+^ adaptive Tregs, and simultaneously drive CD4 T cells to produce IL-4, change the Treg/Th2 balance, block tolerance, and promote the susceptibility of airway inflammation dominated by eosinophils.^332^

The nucleotide-binding oligomeric domain-like receptor family Pyrin domain 3 (NLRP3) inflammasome contains NLRP3, ASC and Caspase-1, which are important constituents of the innate immune system, with critical roles in allergic disorders.^331^ The NLRP3-retinoid X receptor (RXR) axis drives airway epithelial cell apoptosis as well as the production of inflammatory cytokines in the lungs of asthmatic mice.^336^ Further study found that NLRP3 in bone marrow cells promotes the development and progression of AAS in an inflammasome-dependent manner, and RRx-001 (an inhibitor of NLRP3) could significantly decrease inflammatory cell infiltration and mucus secretion in the airway.^337^ PMs can cause acute exacerbation of allergic airway inflammation, activate TLR2/NF-κB/NLRP3 signaling and aggravate allergic airway inflammation.^338^

It was demonstrated that NOD1 and NLRP3 in AR patients are downregulated during the pollen season.^339^ Activation of the NLRP3/gasdermin D/IL-1β signaling pathway mediates macrophage pyroptosis and releases inflammatory mediators to local tissues, which is involved in nasal mucosa inflammation of AR.^340^

### The microbiota plays an essential role in the occurrence and development of allergic diseases

In 1989, Strachan observed that children with more siblings in the family were less likely to develop hay fever or eczema;^28^ children are frequently exposed to allergens, hence the original “hygiene hypothesis” was proposed. This hypothesis is a good explanation for the phenomenon that AAS is significantly more prevalent in developed countries in comparison with underdeveloped countries. From an immunological point of view, it could be understood that the development of immune tolerance to allergens depends on the amount and degree of stimulation by microbial colonization and immune stimulating environmental signals transmitted in early life.^341^

With the rapid development of urbanization and industrialization, excessive use of hygiene products and antibiotics, coupled with changes in diet such as fast food, etc., would decrease microbial diversity in early life,^342^ resulting in impaired immune protection and the destruction of normal microorganisms.^343^ There is increasing evidence that the microbiota is critical for the occurrence and development of allergic disorders.^344^ The human microbiota mainly colonizes the gastrointestinal tract (GIT), with microorganisms also present in other body parts such as the oral cavity, nasal cavity, skin, and respiratory and reproductive tracts.^345^ Symbiotic microorganisms in the GIT and other organs mediate the innate and adaptive immune systems through the gut-lung and gut-skin axes. Reports have shown that many environmental factors influence the colonization, composition and metabolic activities of microbial communities in early life, thereby affecting the immune function of the body and leading to the occurrence of allergic diseases.^346–349^

#### Early-life activities and dysbiosis is tightly associated with allergic diseases

It is well established that microbial colonization starts at birth, and that microbiota composition is affected by related factors such as the prenatal and postnatal environment, which are also critical for the body’s immune function. Multiple factors, including mode of delivery,^350–352^ feeding choice,^353^ and use of antibiotics or not,^354,355^ can alter the composition of the gut microbiota and modulate infant tolerance to different allergens.

The use of antibiotics during pregnancy and the early postpartum period can affect the gut microbiota in normal infants and increase the risk of developing allergic diseases.^356,357^ Mothers exposed to antibiotics during childbirth had significantly lower microbial diversity compared with infants born to antibiotic-free mothers. The microbiota of antibiotic-exposed infants shows reduced amounts of *Bacteroidetes* and *Bifidobacterium*, alongside increased *Proteus* amounts. Studies have shown antibiotic utilization during pregnancy and childbirth is associated with elevated risk of AD and asthma.^358–360^ A study of 14,572 children, 10,220 of whom were administered antibiotics in the initial 2 years of life, revealed that early exposure to antibiotics had tight associations with childhood asthma, AR and AD.^361^ Studies in germ-free laboratory animals further demonstrated an interdependent association of gut microbiota with immune system development.^362–365^

Dysbiosis is tightly associated with allergic disease occurrence, and in some way affects the balance of immune cells in allergic diseases. Most AAS begins in childhood, and HDM, cockroach remains, pet dander, fungi and pollen are the main allergens.^366^ A study found lower abundances of *Lachnospira*, *Veillonella*, *Faecalibacterium*, and *Rothia* in the gut of infants are associated with higher asthma risk, and inoculating these bacteria in germ-free (GF) mice could alleviate airway inflammation and prevent the development of asthma.^367^ Furthermore, decreased abundance of *Bifidobacterium* was found in adult asthmatic patients.^368^ *Haemophilus*, *Moraxella* and *Neisseria spp*. were also observed in the airway microbial composition of asthmatic patients, and *Proteus* was also found in mild cases not receiving inhaled corticosteroids as well as in severe asthma cases. *Actinobacteria and Klebsiella species* were markedly enriched in severe asthma cases in comparison with healthy controls or mild-to-moderate asthma cases.^369^ In AD patients, *Staphylococcus aureus* colonization is an important exacerbating factor in AD pathogenesis, and gut dysbiosis is also considered an important factor in AD pathogenesis. A metagenomic analysis data showed that *S. aureus* constituted approximately 90% of AD skin, leading to dramatically decreased skin microbial diversity.^370^ In animal models, alpha-hemolysin and extracellular vesicles produced by *S. aureus* lead to skin barrier dysfunction and promote atopic skin inflammation.^371–373^ Enterotoxins secreted by *staphylococci* promote allergic skin inflammation by triggering substantial T cell activation, and staphylococcal delta-toxins also cause allergic skin diseases via mast cell activation.^374^ Clinical cohort trials have shown an early reduction in gut microbiota diversity is strongly related to elevated AD risk,^375^ and the presence of gut microbiota subspecies such as *Clostridium Perfringens*, *Clostridium difficile* and *Faecalibacterium prausnitzii* is closely associated with reduced capability of producing short-chain fatty acids (SCFAs), while *L. paracasei* abundance reduces the susceptibility to AD.^376–378^ AR is a respiratory disease that occurs in the upper respiratory tract, which includes the nose and oropharynx. *Firmicutes* and *Actinobacteria* represent key microbiota constituents in the nasal cavity of humans, while *Proteobacteria*, *Firmicutes*, and *Bacteroidetes* represent key phyla in the oropharynx.^379–381^ Microbial diversity in seasonal AR (hay fever) did not decrease, but was instead elevated during allergy season.^382^ *Staphylococcus aureus* is a microorganism involved in perennial (non-seasonal) AR.^383^ The etiology of the elevated incidence of FA remains undefined and may be related to the mode of delivery (natural vs. surgery) or the changes in the microbiome in early life due to antibiotic use even in low amounts.^384–386^

#### The microbiota contributes to the innate and adaptive immune systems in allergic diseases

Human mucosal tissues, such as intestinal mucosa, nasal mucosa, and other surfaces, have regular exposures to complex microbial populations comprising commensal and pathogenic organisms. The host utilizes many molecular mechanisms to mediate mucosal innate immunity for microbial homeostasis. PRRs, including TLRs and NLRs, play major roles in recognizing pathogens and inducing innate immune responses.

Different gut microbiota have different activation pathways, and some gut bacteria, e.g., *Escherichia coli*, *Salmonella typhimurium*, *Klebsiella pneumoniae*, and *Proteus vulgaris*, activate HEK-293 cells through the TLR2 and TLR4 pathways.^387^ Flagellate bacteria, including *Salmonella*, *Listeria*, *Pseudomonas*, and *Escherichia coli*, contain flagellin, which acts by binding to TLR5.^388^ TLR9 functions by recognizing unmethylated CpG motifs, especially GTCGTT motifs, in the DNA of gut bacteria, including *Proteu*s, *Bacteroides* and *Actinobacteria*, as well as *Lactobacillus plantarum*, etc.^389^ TLR signaling stimulates the maturation of innate immune cells, to properly activate APCs and initiate a moderate immune response.^231^ Both exposure to allergic environments and the mother’s allergic status are all factors affecting an infant’s susceptibility to allergic diseases.^390^ Microorganisms are present in the placenta, amniotic fluid and meconium, which are in contact with the fetus in early fetal stage; therefore, the fetus needs to develop immune tolerance during the mother-fetal period to prevent infection.^391^ Natural killers, DCs and macrophages in the endometrium and trophoblasts are already induced during fetal development.^392,393^

In allergic disorders, the microbiome exhibits a crucial function in establishing adaptive and innate immune protection. For instance, children with reduced IgG responses to specific microbial antigens than healthy counterparts are prone to allergic diseases, including asthma, AD and FD.^344,394,395^ In studies of babies, higher AD risk had associations with reduced levels of *Proteobacteria* and elevated innate inflammatory response induced by TLR-4, and *Ruminococcus* decrease was associated with elevated TLR2-dependent innate inflammatory response.^396–398^ FA in early stage is also closely associated with reduced gut microbial abundance.^344^

##### Epithelial cells

Epithelial cells on the nasal and bronchial mucosal surfaces are critical for maintaining the healthy state of respiratory mucosa. Continuous and coordinated ciliary movement enables the removal of foreign invading substances such as pollutants or allergens from surfaces. Epithelial cells also produce different cytokines and chemokines to activate inflammatory cells; meanwhile, hyperactivation of epithelial cells may trigger the onset of several disorders, including asthma and AR.^399^

Epithelial cells recognize PAMPs through innate PRRs, e.g., TLRs and NLRs, and such interactions affect the proliferation of epithelial cells. Microbiota-derived metabolites, including SCFAs, also have effects on epithelial cells. SCFAs, for example, stimulate inflammatory pathways by interacting with GPRs on epithelial cells in the intestine.^400^ P-cresol sulfate (PCS) is a microbial-derived product produced in the gut. PCS selectively reduces CCL20 production by airway epithelial cells due to uncoupling of epidermal growth factor receptor (EGFR) and Toll-like receptor 4 (TLR4) signaling, a pathway that acts distally on airway epithelial cells to reduce allergic airway responses.^401^

##### DCs

DCs, as APCs, are critical for immune responses in contact with commensal microbiota. The presence of SCFA butyrate, an end product of microbial fermentation, stimulates human monocyte-derived dendritic cell (moDC) maturation, increasing IL-10 amounts while decreasing IL-6 and IL-12 levels.^402^ Meanwhile, mice treated with SCFA propionate could produce new myeloid DC precursors with strong phagocytic capability but poor capability of promoting Th2 responses in the lung.

In humans, TLR9 is highly produced by macrophages and plasmacytoid DCs.^403^ The CpG motif is prevalent in bacteria, and the CpG motif also recognizes plasmacytoid dendritic cells (pDC) expressing TLR9 that produce pro-inflammatory cytokines, induce Th1-like immune activation patterns and activate their migration, while CpG-A oligodeoxynucleotides (ODN) induces extremely high levels of IFN-α production and CpG-B ODN induces activation of murine bone marrow-derived DCs to secrete IL-12 and IL-6.^404,405^

In reports assessing AAS disease models in mice and rhesus macaques, macrophage responses to TLR9 activation mainly induce Th1 type immune response, including upregulated TNFα, IL6, IL-12, IL-18, IFN-α and IFN-γ,^406^ resulting in the immune imbalance of Th1/2 cells.^407^

##### Macrophages

The microbiome and associated metabolites, including SCFAs, affect the function of macrophages resident in tissues. In the gut, the SCFA butyrate promotes the anti-inflammatory response of macrophages and induces Treg differentiation by activating its receptor GPR109a, which is essential in inducing tolerance to food. Besides, butyrate exerts anti-inflammatory effects on macrophages by inhibiting IL-6, IL-12 and NO production.^408^ In the lungs of antibiotic-treated mice, macrophages are polarized towards an M2 hypersensitivity phenotype by prostaglandin E2 (PGE 2) produced by commensal fungi.^409^

Lipopolysaccharide (LPS) represents a soluble cell wall constituents in common Gram-negative bacterial organisms.^410^ LPS can interact with complex host systems, including cellular and humoral components of the immune system, to induce the production of multiple immunomodulatory cytokines.^411^ It has been shown that LPS regulates lung inflammation in asthmatic mice via the TLR4 pathway in alveolar macrophages and that different doses of LPS exposure determine the type of inflammatory response.^412^ Improvement of AD by modulation of Th1/Th2 immune system homeostasis with LPS-activated macrophages derived from pantoid epimer (IP-PA1).^413^

##### Mast cells (MCs)

Mast cells (MCs) represent major effector cells in allergic diseases. Increasing evidence suggests that the microbiota can modulate MC function, influence MC activation through direct interactions or secreted metabolites.^414^ In human MCs, co-culture with *Lactobacillus rhamnosus* downregulates high-affinity IgE and histamine H4 receptors, while upregulating IL-8, IL-10, CCL2 and TNF-α.^414^ In murine experiments, *Lactobacillus paracasei* inhibits IgE-mediated MC activation via TLR2. However, the inhibitory effect of *Lactobacillus casei* is mainly through direct cell contact and not dependent on TLR or NOD1/2.^414^

##### Eosinophils

Elevated numbers of eosinophils in AD, AR and eosinophilic allergic asthma are typical symptoms for allergic diseases.^415–418^ Eosinophils act as major effector cells driving innate immunity in allergy and other inflammatory diseases, with an important role in clearing microbes resident in tissues.^419^ Meanwhile, eosinophil function is also regulated by pathogenic microorganisms; for example, *Clostridium difficile* stimulates the release of eosinophil-derived neurotoxins by eosinophils.^420^ However, when eosinophils ingest the probiotic strain *Bifidobacterium*, neurotoxin release is significantly reduced.^420^ Very interestingly, probiotics such as *Lactobacillus fermentum* and *Lactobacillus rhamnosus*, alleviate allergic inflammation involving reduced eosinophil infiltration in multiple mouse models of asthma and AD, although probiotic strains have not been shown to have a direct effect on eosinophils.^421,422^ NLRP12 induces allergic skin inflammation by promoting peripheral DC retention as well as neutrophil migration.^423^

##### Basophils

TLR2 was identified as the primary receptor against *S. aureus*,^244^ and intracellular NLRs also modulate microbial pathogen recognition. Intracellular NLRs, including NOD1, NOD2, NLRP1, NLRP3, NLRP4, and the interferon-inducible protein AIM2 produce a series of inflammatory molecules to resist the invasion of microorganisms.^424^ NOD2 is considered a major player in innate immune response to *S. aureus* in the skin.^425^ Studies have found that NOD2 expression on basophils in the peripheral blood of AD cases is markedly reduced compared with that of healthy people,^426^ and basophils are the main effector cells involved in Th2 polarization in allergic inflammation. *Acinetobacter* in the skin prevents allergic inflammation and is critical for the regulation of Th1/Th2 cell balance and anti-inflammatory responses.^427^

##### Innate Lymphoid Cells (ILCs)

ILCs, a major group of innate immune cells, are present in all parts of the respiratory tract; ILC2 cells are predominantly found in mice, while ILC3 cells are predominantly found in the human respiratory tract.^428,429^ Microbial signaling affects the maturation of ILC tissue-specific functions. *Clostridia* has been shown to stimulate ILC3 to produce IL-22, which helps to strengthen epithelial barrier and reduce intestinal permeability to dietary protein.^430^ ILC3 cells tolerize T-cell response and prevent IL-22 production, leading to a loss of gut bacteria.^409^ Furthermore, when gut macrophages release IL-1β upon microbial insult, ILC3 cells release GM-CSF and induce immune tolerance.^409^ TNF-β produced by ILC3 cells is essential for maintaining gut microbiota homeostasis, and IL-25 is produced by epithelial tuft cells in a microbiota-dependent manner.^409^ The microbiota and its metabolites induce different types of ILCs and regulate their capability of preventing allergic reactions.

##### Tregs

Tregs are important immune regulatory cells, which suppress allergic diseases and have critical functions in controlling the immune response. *Bifidobacterium longum 35624, Clostridium fragilis*, and *Bacteroides fragilis* all induce Tregs in the intestine, whereas other bacteria do not induce Tregs.^431,432^ Activation of PRRs on DCs is a critical mechanism by which gut microbial organisms mediate Treg differentiation.^433^ Recently, it was found that infants with reduced levels of *Bifidobacterium* and *Faecalibacterium* have elevated relative risk of asthma, characterized by elevated amounts of IL-4^+^ Th2 cells and reduced Treg levels,^434^ attenuating the adaptive immune response. In IgE-induced FA studies, dysbiosis affected the differentiation of Tregs, resulting in an imbalance of Treg and Th2 cells and leading to allergic diseases (Fig. 3).^435,436^

## Treatment

At present, the treatment of allergic diseases mainly includes five aspects: management and therapeutic education for patients and allergen avoidance, traditional pharmacotherapy, allergen immunotherapy, biologics administration and other therapies.

### Management and therapeutic education for patients and allergen avoidance

Daily management of patients with allergic diseases and educational intervention plays an essential role in managing difficult-to-treat allergic cases. It is frequent and may lead to treatment failure due to poor adherence to the prescribed treatment.^437^ Ensuring patient education and confidence in prescribed drug is urgently required, to achieve disease control. For example, it is necessary to inform patients regarding the correct use of intranasal spray and other pharmaceutical preparations.^438^ What’s more, psychosomatic aspects can also contribute to complementing topical and systemic therapies.^439^ Furthermore, it is also a critical issue in allergic disease management to combine the limited resources and increased digitalization. For example, mobile applications can monitor symptoms, drug use, and quality of life timely with higher efficiency, e.g., the Allergy Diary.^440,441^

Avoiding allergens or minimizing exposure to allergens is the first step of treatment. However, since the pathogenic allergens of different patients are often different, mainly including food allergens, aeroallergens, contact allergies, and allergens in the environment that are frequently unknown, the strategy of avoiding allergens on the basis of an allergy diagnosis is often challenging.^438^ Therefore, teaching patients how to avoid contacting with allergens may help achieve the best treatment effect. For example, the current standard of FA care is to avoid allergens, because there is no Food and Drug Administration (FDA) approved treatment for FA at present.^442^ As for AR or AD, due to their multifactorial etiologies, elimination of food or environmental allergens represents an adjuvant treatment to pharmacotherapy; thus, complete remission may not be expected after mere allergen elimination.^443^ Other allergen avoidance measures such as environmental intervention and use of protective devices are available to reduce exposure to causative substances for maximum effectiveness, which apply to all forms of asthma aggravated by the working environment.^444^ It is noteworthy that determining the individual’s relevant triggers for allergic diseases may be more difficult than diagnosing the disease itself.^439^ To sum up, allergic diseases normally need drugs for treatment.

### Traditional pharmacotherapy

Traditional pharmacotherapy is currently an efficient and rapid treatment method, which helps improve the symptoms of most patients and enhance their quality of life. At present, there are many kinds of drug treatments for allergic diseases, with good therapeutic effects (Table 2).

**Table 2:** Different Traditional pharmacotherapies and their mechanism of action and clinical application of drugs

Traditional pharmacotherapy		Mechanism	Drugs	Stage	References
H1-antihistamine	First-generation	Serve as neutral receptor antagonists or inverse agonists of the histamine H1 receptor, can block the action of histamine	Chlorpheniramine	Marketed	^448^
			Diphenhydramine	Marketed	^448^
			Hydroxyzine	Marketed	^448^
			Doxepin	Marketed	^448^
	Second-generation		Loratadine	Marketed	^446^
			Cetirizine	Marketed	^446^
			Fexofenadine	Marketed	^446^
			Rupatadine	Marketed	^446^
			Bilastine	Marketed	^446^
			Astemizole	No longer approved	^447^
			Terfenadine	No longer approved	^447^
	Third-generation		Desloratadine	Marketed	^449^
			Fexofenadine	Marketed	^449^
			Levocetirizine	Marketed	^446^
Corticosteroids	Intranasal corticosteroids (INCS)	Inhibit a variety of inflammatory genes, including cytokines, inflammatory enzymes, adhesion molecules and inflammatory mediator receptors	Beclomethasone	Marketed	^438^
			Budesonide	Marketed	^438^
			Ciclesonide	Marketed	^438^
			Fluticasone propionate	Marketed	^438^
			Fluticasone furoate	Marketed	^438^
			Mometasone furoate	Marketed	^438^
			Triamcinolone acetonide	Marketed	^438^
	Topical corticosteroids (TCS)		Prednicarbate	Marketed	^439^
			Futicasone	Marketed	^439^
			Mometasone	Marketed	^439^
	Systemic corticosteroids (SCS)		Methylprednisolone	Marketed	^439^
Combination of H1-antihistamine and nasal corticosteroids		Can block the main cause of direct response and reduce inflammation through significantly different mechanisms	Azelastine hydrochloride [HCl] and fluticasone propionate	Marketed	^457^
Leukotrienes (LTS)		5-LO inhibitors prevent the synthesis of leukotriene A4 by inhibiting the catalytic activity of 5-LO, LTRAs block the cysteinyl LT (cysLT) receptor type 1 directly	Pranlukast	Marketed	^458^
			Montelukast	Marketed	^458^
			Zafirlukast	Marketed	^458^
Other drugs	Mast cell stabilizer	Inhibit the degranulation of allergic mediators	Sodium cromoglicate	Marketed	^463^
	Chromones	Preventive antiallergic drugs	Cromolyn sodium	Marketed	^438^
	Phosphodiesterase 4 inhibitors	Promote apoptosis by reducing the anti-apoptotic protein bcl-2, which can inhibit neutrophils and eosinophils in vitro	Crisaborole	Marketed	^439^
	Topical calcineurin inhibitors (TCI)	An inhibition of proinflammatory cytokine production by T cells and mast cells, reveal antipruritic effects which attributes to a specific effect on TRPV1 neurons in the skin	Tacrolimus	Marketed	^469^
			Pimecrolimus	Marketed	^469^
	Other immunosuppressants	Inhibit the proliferation and differentiation of immune cells and the differentiation of T cells	Azathioprine	Marketed	^462^
			Cyclosporine A	Marketed	^462^
	Bronchodilators	Prevent and relieve bronchoconstriction	Long‐acting muscarinic antagonist (LAMA)	Marketed	^466^
			Long‐acting beta‐agonist (LABA)	Marketed	^466^

Website for information inquiry on Traditional clinical drugs https://www.yaozh.com/. Drugs that have been approved for listing are marked with “Marketed”

#### H1-antihistamines

H1-antihistamines, which serve as neutral receptor antagonists or inverse agonists of the histamine H1 receptor, can block the action of histamine.^445^ According to brain H1 receptor occupancy (H1RO, an indicator of antihistamines), H1‐antihistamines can be divided into the non-sedating, less-sedating and sedating groups, whose diverse chemical structures, pharmacokinetic features and potential for drug-drug and drug-food interactions make them different. First-generation H1-antihistamines have not been well studied, and because of their adverse effects, particularly sedation, they should be avoided and not recommended for use.^438^ New second-generation H1-antihistamines have great efficacy and safety profiles, as well as good tolerance.^446^ The development of second-generation H1-antihistamines occurred in the 1980s, revolutionizing allergy therapy due to no or only minimal sedative potential, including the less-sedating oral H1-antihistamines and non-sedating H1-antihistamines.^447^ However, because of cardiotoxic side effects, two early second-generation H1-antihistamines, i.e., astemizole and terfenadine, have now been withdrawn from the market.^447,448^ Third-generation oral antihistamines have improved efficacy, safety, and pharmacological and pharmacokinetic features, representing suitable candidates for the treatment of seasonal or perennial allergies, which may improve the allergic symptoms of patients.^449^

#### Corticosteroids

Corticosteroids were considered the most effective therapeutic approach for atopic disorders in the past, and could control virtually all cases of allergic diseases at high dose.^450^ The main role of corticosteroids is to inhibit a variety of inflammatory molecules such as cytokines, pro-inflammatory proteins, adhesion molecules and inflammatory receptors, which explain their high efficacy in complex inflammatory diseases. Corticosteroids are divided into intranasal corticosteroids (INCS), topical corticosteroids (TCS) and systemic corticosteroids (SCS).

INCS are a well-established first-line therapeutic option for adults and children with persistent or moderate-to-severe symptoms, decrease inflammation associated with AR and alleviate nasal and ocular symptoms.^438,451^ Their efficiency is more obvious than that of oral or intranasal antihistamines and antileukotrienes. In addition, INCS are comparable to the combination of antihistamines and antileukotrienes.^452^ Mechanistically, INCS exert local anti-inflammatory effects on nasal mucosal cells. New-generation inhaled corticosteroids for asthma show enhanced anti-inflammatory effects with minimal adverse effects. Undoubtedly, inhaled corticosteroids (ICS) have revolutionized asthma therapy, and are currently considered the first-line therapeutic option for all chronic asthma cases.

Topical corticosteroids (TCS) induce fewer systemic side effects, especially the recently developed topical steroids that have short half-lives, and are the first-line anti-inflammatory approach in AD.^439,453^ Studies have shown that topical corticosteroids used as adjunctive therapy alongside dupilumab may provide additional benefits.^454^ Systemic corticosteroids (SCS), one of the groups of drugs available for systemic anti-inflammatory therapy, are required for AD not sufficiently controllable with adequate topical therapies and UV light therapy. Although SCS have rapid effects, their use should be limited to 1-2 weeks because of significant risk of severe long-term side effects, as observed with methylprednisolone.^439^

#### Combination of H1-antihistamines and nasal corticosteroids

Conceptually, it is meaningful to use both nasal corticosteroids and local nasal antihistamines at the same time, because such treatment can block the main cause of direct response and reduce inflammation through significantly different mechanisms.^455^ A study addressed the possibility that a combination of azelastine and fluticasone (both as nasal spray) can confer significant clinical benefits in individuals with seasonal allergic rhinitis (SAR) in comparison with any drug alone.^456^ It has been shown that the nasal symptoms of SAR are more significantly relieved by the fixed-dose combination (FDC), containing an intranasal antihistamine (azelastine hydrochloride [HCl]) and a corticosteroid (fluticasone propionate) compared with placebo or monotherapy, and could also alleviate the nasal symptoms of perennial allergic rhinitis (PAR) more substantially than fluticasone monotherapy.^457^

#### Leukotriene receptor antagonists

Leukotrienes (LTS) are indispensable for the pathogenetic mechanisms of allergic inflammation, so inhibiting LTS represents an effective and feasible strategy in the treatment of allergic diseases, including AAS, AR, and AD. Leukotriene receptor antagonist (LTRA) is an additional treatment option for asthmatics and AR cases. In addition, some leukotriene receptor antagonists are currently used clinically for exercise-induced bronchoconstriction.^458^ Leukotriene is an important lipid mediator in asthma-related research.^459^ Leukotriene receptor antagonists improve small airway function and reduce airway inflammation in the treatment of AAS.^460^ In Europe, LTRAs have been approved by European Medicine Agency(EMA) only for the treatment of asthma and AR.^438^ Studies have shown that early intervention with a 4-week anti-leukotriene course is also beneficial for some pollen allergies.^461^ LTRAs are not known to cause significant congenital malformations or adverse perinatal outcomes in pregnancy safety studies. Some reports point out that LTRAs may be considered in second-line treatment of pregnant women if better treatments fail.^462^

#### Other drugs

Other drugs can also help treat allergic diseases. Mast cells can regulate angiogenesis, tissue inflammation and repair. The cells play an important role in innate and adaptive immune response, immune tolerance, and host defense. Therefore, mast cells are essential for allergic reactions. Mast cell stabilizer such as sodium cromoglicate is targeted at mast cells, which can inhibit the degranulation of allergic mediators, thus preventing allergic diseases. The side effects of sodium cromoglicate are less, and it has been clinically approved for the treatment of asthma and AR. However, the therapeutic time window of mast cell stabilizer is relatively narrow. The patient needs to be given the drug immediately before the allergen stimulation, so that the drug can play a stable and effective role.^463–465^

Some drugs are used for symptomatic treatment of allergic diseases. For example, most chromones can be administered as monotherapy for local symptoms. They are relatively safe drugs, but with low efficacy.^438^ Theophylline, a Phosphodiesterase 4 (PDE4) inhibitor, is another drug that promotes apoptosis by reducing the anti-apoptotic protein Bcl-2, which inhibits neutrophils and eosinophils in vitro. It was also noted that theophylline inhibits reactive oxygen species accumulation by neutrophils and reduces neutrophil chemotaxis.^466^ At present, there are four PDE4 inhibitors approved for treating human diseases, including AD and bronchial asthma.^467^ However, considering the associated systemic adverse effects, topical administration of inhaled PDE4 inhibitors might represent a promising alternative instead of systemic utilization, although these drugs are not recommended to use before or during pregnancy.^462,468^

Topical calcineurin inhibitors (TCIs) have substantial anti-inflammatory effects, inhibiting the biosynthesis of proinflammatory cytokines by T cells and mast cells, as well as antipruritic effects that are attributed to specific effects on skin Transient receptor potential vanilloid 1 (TRPV1) neurons.^439^ TCIs are especially useful in individuals requiring long-term treatment, and two of them have been approved for topical AD therapy; nevertheless, reports assessing the application of topical calcineurin inhibitors during pregnancy are limited.^469^ It is important to note that other immunosuppressants, including azathioprine and cyclosporine, do not induce congenital malformations; besides, cyclosporine is considered as first-line drug for long-term treatment of diseases.^462,470^

Bronchodilators, which are significant for preventing and relieving bronchoconstriction, including long‐acting muscarinic antagonist (LAMA) and long‐acting beta‐agonist (LABA) addition to ICS, sometimes have adverse effects, including tremors, palpitations and tachycardia.^466,471,472^

In general, H1-antihistamines are usually safe and widely used for the treatment of various allergic diseases, but some patients experience adverse effects, such as cardiotoxicity, central depression and anticholinergic effects. In addition, there are individual differences in the efficacy of antihistamines in clinical practice.^473^ INCS are effective in combating nasal and ocular symptoms and improving quality of life, and are safe for short-term use, but long-term safety data are lacking. Although generally well tolerated, adverse events can be observed that may lead to serious ocular complications.^474^ As for TCS and SCS, long-term continuous use of corticosteroids may result in local and systemic toxicity risks. LTRAs are well tolerated, non-hormonal anti-inflammatory drugs. LTRAs are primarily employed for long-term control treatment of patients with mild asthma and comorbid AR. It has also been shown that LTRAs are not associated with the risk of major congenital malformations and can be safely used during pregnancy to treat asthma.^475^ Nevertheless, the anti-inflammatory effect of LTRAs is not as strong as that of corticosteroids, so they are often used in combination with inhaled corticosteroids to enhance their efficacy in clinical practice. However, Some experiments have shown that LTRAs and oral H1-antihistamines have comparable effects in AR.^438^ In short, based on the side effects of these traditional drugs, other therapies are emerging as well, such as allergen immunotherapy (AIT) and biologics, etc.

### AIT

AIT constitutes the sole disease-modifying therapy for patients with IgE-mediated inhalation allergic diseases.^476^ Compared with traditional pharmacotherapy, the advantage of AIT is that it is a medical intervention that can limit the natural process of the disease.^477^ The purpose of this therapy is to reduce the symptoms of allergic diseases by inducing tolerance to allergens.^438^ AIT is based on the administration of allergens that cause a given disease. Through long-term repeated exposure to specified doses of allergens, it can change the immune response and induce protective immunity, so that patients may tolerate future allergen exposure.^478,479^ In addition, this therapy reduces the long-term treatment cost for patients and the economic burden of allergy. From the perspective of public health, AIT plays a crucial role in allergy management.^477^

#### Application of AIT in allergic diseases

In allergic diseases, immune dysfunction is the key pathogenic factor, so the concept of inducing immune tolerance has gradually become one of the goals set for preventing and treating allergic diseases.^477^ In the routine use of AIT, it is recommended to take a 3-year course of treatment to achieve long-term efficacy, and long-term clinical benefits are achievable after stopping treatment.^476,480^ In adolescents and adults, AIT can be used to treat moderate-to-severe rhinitis and moderate asthma. In children, AIT can prevent rhinitis cases from further developing asthma symptoms.^481^ Researchers analyzed AR progress and changes in asthma symptoms after HDM allergen immunotherapy. The results showed that treatment of AR patients with HDM allergy drugs could indeed reduce the overall incidence rate of asthma, improve asthma symptoms and slow down the progression of asthma.^482,483^ Many experiments have revealed that AIT markedly reduces the symptoms in patients, changes the disease process, and improves the quality of life in allergic individuals.

#### Therapeutic principles of AIT in allergic diseases

The tolerance induced by AIT is related to changes in allergen-specific memory T and B cells as well as allergen-specific IgE and IgG antibody amounts. In addition, AIT has some impact on the activation threshold of mast cells, basophils and dendritic cells.^438^ Some cells utilize interleukin-10 (IL-10), IL-35, transforming growth factor-β (TGF-β), IL-10R, TGF-βR, cytotoxic T lymphocyte-associated antigen 4 (CTLA4), programmed cell death protein 1 (PD1) and other inhibitors to directly or indirectly alleviate the anaphylactic environment.^484^ Studies have shown that AIT regulates the follicular helper T (TFH) cell-to-follicular regulatory T (TFR) cell balance in allergic cases. The decrease and functional defect of TFR cells are associated with AR.^485^ Studies have also shown that AIT reduces the expression of CD23 on switched memory B cells, which has a positive correlation with the clinical efficacy of AIT in AR.^480^

#### Biomarkers in AIT can predict or monitor immune responses to determine efficacy as early as possible

In AIT, biomarkers can reflect some clinical or laboratory characteristics of the immune process, which is essential for monitoring the health status of patients at all times. However, there is a lack of validated genetic or blood markers that could help predict or monitor the efficacy of AIT at the individual patient level.^481^ In recent years, researchers have been screening for biomarkers to detect the success of AIT. Many technologies are expected to be used to examine these biomarkers, including genomics, transcriptomics, immunology, lipidomics, metabolomics, microbiology, epigenetics and proteomics.^478^ According to the mechanism of anaphylaxis and the development and application of the above technologies, relatively reliable candidate biomarkers for immune detection have been reported, including immunoglobulins (allergen-specific IgD, IgE and IgG4) and some cytokines or chemokines (IL-4, IL-13, IL-9, IL-25, IL-33 and TSLP). In addition, candidate biomarkers can also include specific genes or immune-related cells such as cytokine and immunoglobin transcripts, myeloid cells, innate lymphoid cells (ILC), T cells and B cells.^478^

#### The drug delivery route affects the quality of AIT

In AIT, different drug delivery routes show different side effects and final efficacy. Conventional AIT includes subcutaneous immunotherapy (SCIT) and sublingual immunotherapy (SLIT).^476,486^ Traditional SCIT has certain shortcomings that cannot be ignored. It requires frequent medical treatment and multiple injections, and in some rare cases, life-threatening allergic reactions and adverse events may occur.^487^ The typical local reaction in SCIT is redness and swelling at the injection site, and more serious systemic reactions include sneezing, nasal congestion and urticaria. Some serious symptoms often occur within 30 minutes, so individuals are usually allowed to leave the hospital 30 minutes after completing the injection.^481,488^ SLIT has become a particularly attractive alternative to AIT because of its rather easy administration. It usually involves allergen drops or tablets, which can be administered at home.^489^ Some new routes of AIT administration are also being developed in order to improve the safety and convenience of patients and maintain or even obtain better curative effects.^476,477,490^ Oral immunotherapy (OIT) is another SCIT option. OIT seems to have no effects on most respiratory allergens because they are easily digested in the gastrointestinal tract. Therefore, OIT is mainly used for anti-digestive food allergens, including milk, eggs, peanuts and some wheat.^479^ Studies have shown that OIT for IgE-mediated FA desensitizes patients who are at risk or have experienced severe allergies to peanuts, eggs and milk.^477^ Intralymphatic immunotherapy (ILIT) is also another better choice for SCIT. This method involves intralymphatic administration, because lymph nodes contain a large number of immune cells, so their direct contact with allergens would be faster than in SCIT and produce stronger protective IgG antibodies and immune regulation at the same time.^479,491^ In addition, studies have proposed epicutaneous immunotherapy (EPIT), which aims to cause fewer systemic side effects when administered through the epidermis.^479^

In conclusion, the current goal of improving AIT is to shorten the treatment time, improve its efficiency in order to promote the absorption and presentation of allergens, reduce side effects to improve safety, improve patient compliance, and ultimately significantly increase the utilization of this treatment method.^492^

### Application of biologics in allergic diseases

**Fig. 6:**
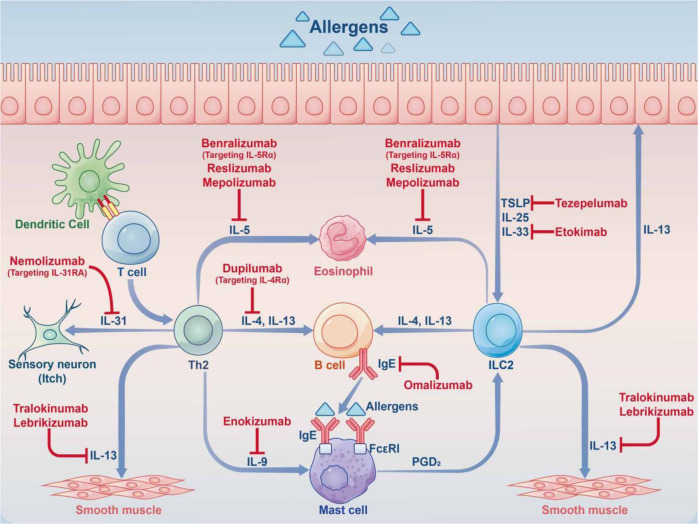
Application of biologics in allergic diseases. Multiple cell interactions trigger allergic reactions. Therefore, treatment with biologics aims to target the cytokines produced by various cells, with a potential impact on the interaction between cells. Some biologics exert their effects by targeting IgE, IL-5, IL-4, IL-13, IL-31, IL-9, IL-33, and TSLP, among others

New studies on allergic diseases are underway, especially the development and application of new biologics.^441^ So far, many biologics targeting Th2/1/17 inflammatory biomarkers are available, many of which are clinically applied.^493^ Identifying new and reliable biomarkers and clarifying novel molecular mechanisms that persist in specific reactions have become important research directions.^438^ We summarized some existing biologics according to differences in the mechanisms of action and target sites, providing a certain reference for follow-up studies of biologics (Table 3, Fig. 6).

**Table 3:** Targets and clinical stages of biologics

Biologic	Description	Clinical stage	References
Omalizumab	Recombinant humanized anti-IgE antibody	Marketed	^441^
Mepolizumab	IgG1 antibody against IL-5	Marketed	^496^
Reslizumab	Anti-IL-5 IgG4 antibody	Marketed	^496^
Benralizumab	An IL-5Rα-directed cytolytic monoclonal antibody	Marketed	^496^
Dupilumab	Fully humanized antibody that targets IL-4Rα subunits	Marketed	^500^
Tralokinumab	IgG4 monoclonal antibody targeting IL-13	Marketed	^500^
Lebrikizumab	Inhibit the dimerization of IL-13Rα1 and IL-4Rα	Phase III	^500^
Nemolizumab	Humanized monoclonal antibody targeted to block IL-31 receptor A	Marketed in Japan	^501^
Enokizumab	A humanized immunoglobulin G1k anti-IL-9 mAb	Phase II	^516^
Baricitinib	Small molecule that inhibit JAK1 and JAK2	Marketed	^501^
Tofacitinib	Inhibit JAK1 and JAK3	Marketed	^501^
Upadacitinib	A selective JAK1 inhibitor	Marketed	^501^
Ruxolitinib	Small molecule JAK1/2 inhibitor	Marketed	^505^
Teglarinad Chloride	Inhibitors targeting IKK complex in NF-κB pathway	No application	^542^
Disulfiram	Inhibitors targeting UPS in NF-κB pathway	No application	^542^
TPCA-1	Small molecule IKK-β inhibitor	No application	^218^
AS602868	Small molecule IKK-β inhibitor	No application	^218^
Idelalisib	PI3Kδ kinase inhibitor	Marketed	^542^
Alpelisib	PI3K inhibitor, inhibiting PI3Kα (PIK3CA) activity	Marketed	^542^
Copanlisib	PI3K inhibitor, inhibiting PI3Kα and PI3Kδ	Marketed in the USA	^542^
Duvelisib	Selective PI3K δ/γ inhibitor	Marketed	^542^
Rapamycin	mTOR protein specific inhibitor	Marketed	^505^
SAHM1	Inhibit the formation of classical Notch transcription complex	No application	^510^
DAPT	γ- Secretase inhibitor, inhibit Notch 1 signaling	No application	^510^
Avasimibe	Inhibit the Wnt/β-catenin signaling pathway	No application	^511^
Resiquimod (R848)	Agonist of Toll-like receptors 7/8 (TLR7/TLR8)	No application	^512^
GSK2245035	Toll-like receptor 7 agonist	Phase II	^513^
Tezepelumab	Monoclonal antibody against TSLP	Marketed in the USA	^514^
Etokimab	IgG1 antibody can neutralize IL-33	Phase II	^515^
GBR830	An antibody targeting OX40	Phase II	^500^
AMG853	A CRTH2 antagonist reduces Th2-mediated inflammation	Phase II	^189^
OC000459	A CRTH2 antagonist reduces Th2-mediated inflammation	Phase III	^189^
BI671800	A CRTH2 antagonist reduces Th2-mediated inflammation	Phase I	^189^
N6022	Small molecule inhibitor of GSNOR	Phase II	^519^

Website for information inquiry on biologics https://www.yaozh.com/. Biologics that have been approved for listing are marked with “ Marketed”, and unlisted biologics are inquired about their clinical research stage in allergic diseases. “ No application” indicates that no clinical study on the application of this biological agent to treat allergic diseases has been found

#### Targeting IgE

Targeting IgE molecules is one of the most important methods for nipping allergic reactions in the body with lasting effects. Anti-IgE antibody treatment markedly reduces the serum amounts of free IgE molecules in allergic individuals, thus exerting a certain effect.^494^ Recombinant humanized anti-IgE antibodies, such as omalizumab, have been approved by the FDA for severe asthma, substantially improving clinical symptoms in patients with poor disease control.^441,495^

#### Targeting IL-5

Eosinophils are critical in asthma pathogenesis, and airway eosinophil inflammation is related to the severity of asthma. Biologics, including mepolizumab, reslizumab and benralizumab, have been developed to target IL-5 or IL-5Rα and then affect the survival and differentiation of eosinophils. Studies have shown that blocking the IL-5 receptor could indeed alleviate severe eosinophilic asthma and severe uncontrolled asthma.^496–498^

#### Targeting IL-4/IL-13

IL-4 and IL-13 represent key driving factors in type 2 immune diseases. For example, dupilumab was approved by the FDA and the EMA (EU) for adults with moderate-to-severe AD. Dupilumab is a fully humanized antibody that targets IL-4Rα subunits, thus inhibiting the IL-4/IL-13 axis. These two cytokines induce IgE production in B cells, goblet cell metaplasia, mucus production, basement membrane thickening and fibrosis. A recent experiment showed that blocking the IL-4/IL-13 pathway alleviates glucocorticoid-dependent severe asthma, moderate-to-severe uncontrolled asthma and AD. Biologics also significantly alleviate AR symptoms, especially nasal symptoms.^441,496,497,499^ Many biologics have been developed to target IL-4 and IL-13, inhibiting the dimerization of IL-13Rα1 and IL-4Rα or directly targeting the IL-4Rα subunits to play a role.^499–502^ More and more biologics that block IL-4 and IL-13 activities have been shown to alleviate nasal symptoms in patients with uncontrolled asthma and AR.^503^

#### Targeting IL-31

Monoclonal antibodies, including nemolizumab, a humanized monoclonal antibody targeting IL-31 receptor A (IL-31RA), relieve itching symptoms and IL-31 signal transduction in the pathogenesis of AD. In mice, IL-31 and its receptor IL-31RA are involved in AD-induced pruritus. In another animal experiment, the monkey scratch model, it was found that the scratch induced by IL-31 is significantly alleviated after a single injection of nemolizumab.^501,504^

#### Targeted signaling pathways

Some biologics target signaling pathways, including baricitinib, tofacitinib, upadacitinib and ruxolitinib, which target JAK/STAT signaling. Targeting JAK/STAT signaling is critical for T cell activation, and could also block the downstream pathways of many important cytokine receptors. T cells are essential for atopic inflammation. The JAK protein in cells activates STAT protein dimerization and transfer to the nucleus, thereby increasing the gene expression of inflammatory mediators. Therefore, JAK/STAT pathway suppression represents an effective and feasible therapeutic approach in AD.^500,501,505^ Asthma is often accompanied by inflammation, and many pro-inflammatory chemokines, cytokines, adhesion molecules, airway mucins, growth and angiogenesis factors are upregulated through Rel/Nuclear Factor-κB (NF-κB) transcription factor family.^218,506^ So asthma and NF-κB mediated signal transduction is inextricably linked, and a series of NF-κB signaling intermediate inhibitors have been produced, e.g., DNA oligonucleotides and DNA-peptide molecules acting as NF-κB bait sequences; in addition, small molecule inhibitors and some proteasome inhibitors affect NF-κB signal transduction.^218^ For example, small molecule inhibitors such as TPCA-1 and AS602868 inhibit IκB kinase (IKK)-β to block NF-κB signaling. In addition, proteasome inhibitors can block NF-κB signal transduction by targeting the 20 S proteasome, ultimately regulating eosinophil function.^218,507^ Some biologics regulate the signal cascade by inhibiting PI3K or any downstream target, because PI3K-mediated signals pass through IKKα phosphorylation, and protein kinase B (PKB, AKT) phosphorylation directly activates IKK, which then enters NF-κB signal cascade reaction pathway. For example, idelalisib, alpelisib, copanlisib and duvelisib are PI3K inhibitors.^508^ The mechanistic target of rapamycin (mTOR) pathway is also considered a signaling pathway regulating innate and adaptive immune cells. It was found that rapamycin targeting mTOR inhibits eosinophil differentiation and reduces allergic airway inflammation in mouse models.^505,509^ Notch signaling is also associated with the pathogenesis of allergic airway inflammation. Notch is necessary to maintain Th1 and Th2 programs. As a biological agent, stapled α-helical peptide derived from mastermind-like 1 (SAHM1) targets Notch to effectively reduce the inflammatory symptoms of mice with experimental allergic airway inflammation and accelerate recovery. In addition to being related to T cells, Notch also is critical for the differentiation of lung organs and alveoli. For example, in a mouse asthma model, intranasal administration of γ-secretase inhibitors (GSIs) could block the Notch signaling pathway and reduce allergic pulmonary edema.^510^ Avasimibe (Ava), as a specifically targets acetyl-CoA acetyltransferase 1 (ACAT1) inhibitor, has been proven to alleviate the disruption of the airway epithelial barrier by inhibiting the Wnt/β-catenin signaling pathway. It has good safety and may be a promising drug for the clinical treatment of AAS.^511^

#### Other biologics

Currently, the use of new therapeutic targets is also being explored. For example, Toll-like receptors are involved in the activation of innate immunity in the respiratory mucosa. Some agonists can induce cytokines, including TNF-α, IL-6 and IFN-α; such biologics are well tolerated and may not cause systemic immune activation.^512,513^ In the study of AAS, due to its complex pathogenesis, certain new biological targets have attracted attention from researchers, e.g., epithelial cell-derived cytokines (IL-1, IL-33, IL-25 and TSLP).^496,503,514,515^ In addition, IL-9 is also associated with allergic diseases. In the study of asthma, mouse models overexpressing IL-9 show enhanced eosinophilic airway inflammation, IgE production and mast cell proliferation. Therefore, biologics targeting IL-9 have also been designed.^516^ There are also antibodies that target the costimulatory molecule OX40 (CD134), which is critical for T cell expansion.^500^

Chemoattractant receptor-homologous molecule expressed on TH2 cells (CRTH2), also known as prostaglandin D2 receptor 2, is a 7-transmembrane G protein-coupled receptor expressed by Th2 cells, eosinophils and basophils. The CRTH2 receptors are involved in inducing the migration and activation of Th2 lymphocytes, eosinophils and basophils. At the same time, it is also involved in the up regulation of adhesion molecules and the release of proinflammatory Th2 cytokines such as IL-4, IL-5 and IL-13. Therefore, it plays a certain role in the course of allergic diseases. It has been proved that the number of CRTH2-positive cells is related to the severity of asthma. At present, some CRTH2 antagonists, such as AMG853, OC000459 and BI671800, have been developed to treat asthma, AD and AR. These CRTH2 inhibitors reduce the Th2-mediated inflammatory response by blocking the activation of mast cells, basophils and eosinophils.^189,517,518^

Nitric oxide (NO) is an important signal molecule in many physiological processes, such as maintenance of vascular tone and endothelial barrier function, immune defense and apoptosis. NO can also regulate cell function through post-translational modification of proteins. NO can react with glutathione to form S-nitrosoglutathione (GSNO), which effectively transduces NO signal. GSNO concentration can be regulated by GSNO-reductase (GSNOR), which provides the “brake” for signal transduction. There is evidence that GSNOR polymorphisms increase the expression of GSNOR and are associated with an increased risk of asthma. Inhibition of GSNOR can lead to the preservation of endogenous GSNO and limit eosinophilic inflammation, mucus production and airway hyper reactivity (AHR). N6022 is the first small molecule inhibitor of GSNOR in the treatment of asthma. Some studies have shown that in the mouse asthma model, N6022 significantly reduces airway hyperreactivity and shows a strong anti-inflammatory effect.^519–521^

Biologics-targeted therapies target the endotypes of allergic diseases based on pathogenesis, reducing the occurrence of adverse events and improving the efficiency of treatment, and are considered promising therapeutic approaches. However, many biologics have not been developed far enough and have not been better evaluated. The response to treatment varies greatly from individual to individual. Therefore, the dose and duration of treatment need to be better defined and the details need to be further optimized. In addition, the relatively expensive price of biologics also limits their application to some extent.^522–524^

### Other therapies

Different treatment options have varying degrees of side effects; thus, a growing number of alternatives therapies have also been developed. In addition to the aforementioned treatment methods, other therapies also offer different therapeutic effects in the treatment of allergic diseases, including antibacterial and antimycotic therapies, phototherapy, early introduction therapy, circadian regulation therapy and so on.

The microbiota and allergic diseases are closely related. For example, *Staphylococcus aureus* is the main cause of AD, and *Malassezia furfur* is also associated with skin immune response and barrier function. Therefore, antibacterial and antimycotic therapies have also become an option for treating allergic diseases.^439^ Phototherapy has immunosuppressive and immunomodulatory effects, which can inhibit the effects of anaphylaxis and histamine release triggered by mast cell antigens. Therefore, rhinophototherapy is considered a promising non-invasive alternative treatment option for perennial or seasonal AR cases, especially low-level laser therapy (LLLT).^525,526^ However, it has been demonstrated that photochemotherapy has certain carcinogenicity. In addition, in AD, oral psoralen plus ultraviolet A (PUVA) as a type of phototherapy also has many side effects. Therefore, PUVA for AD treatment has been abandoned to a large extent, and it is recommended to apply combination therapy.^439^ In patients with FA, early consumption is more beneficial than delayed administration. A trial showed that early introduction of eggs combined with appropriate eczema treatment is practical, and may effectively reduce the odds of egg allergy in high-risk infants.^527^ One of the important research fields to further examine new methods to prevent or treat modern allergic diseases is to understand the relationship between circadian biology and allergy; correspondingly, it is suggested to develop a lifestyle in which the endogenous biological clock is consistent with the environmental cycle, or undergo appropriate therapeutic interventions to prevent or treat allergic disease in the future.^528^
